# 8-Cl-Ado and 8-NH_2_-Ado synergize with venetoclax to target the methionine-MAT2A-SAM axis in acute myeloid leukemia

**DOI:** 10.1038/s41375-024-02222-w

**Published:** 2024-04-20

**Authors:** Jiamin Guo, Ralf Buettner, Li Du, Zhenlong Li, Wei Liu, Rui Su, Zhenhua Chen, Yuan Che, Yi Zhang, Rui Ma, Le Xuan Truong Nguyen, Roger E. Moore, Pathak Khyatiben, Min-Hsuan Chen, Pirrotte Patrick, Xiwei Wu, Guido Marcucci, Lili Wang, David Horne, Jianjun Chen, Yanzhong Yang, Steven T. Rosen

**Affiliations:** 1 Irell & Manella Graduate School of Biological Sciences, Beckman Research Institute, City of Hope, Duarte, CA USA; 2 Department of Hematology and Hematopoietic Cell Transplantation, City of Hope, Duarte, CA USA; 3 Department of Systems Biology, Beckman Research Institute, City of Hope, Duarte, CA USA; 4 Department of Cancer Genetics and Epigenetics, Beckman Research Institute, City of Hope, Duarte, CA USA; 5 Department of Hematologic Malignancies Translational Science and Division of Leukemia, City of Hope, Duarte, CA USA; 6 Integrated Mass Spectrometry Shared Resource, City of Hope, Duarte, CA USA; 7 Cancer & Cell Biology Division, Translational Genomics Research Institute, Phoenix, AZ USA; 8 Integrative Genomics Core, Beckman Research Institute, City of Hope, Duarte, CA USA; 9 Department of Cancer Biology and Molecular Medicine, Beckman Research Institute, City of Hope, Duarte, CA USA

**Keywords:** Acute myeloid leukaemia, Targeted therapies

## Abstract

Targeting the metabolic dependencies of acute myeloid leukemia (AML) cells is a promising therapeutical strategy. In particular, the cysteine and methionine metabolism pathway (C/M) is significantly altered in AML cells compared to healthy blood cells. Moreover, methionine has been identified as one of the dominant amino acid dependencies of AML cells. Through RNA-seq, we found that the two nucleoside analogs 8-chloro-adenosine (8CA) and 8-amino-adenosine (8AA) significantly suppress the C/M pathway in AML cells, and methionine-adenosyltransferase-2A (MAT2A) is one of most significantly downregulated genes. Additionally, mass spectrometry analysis revealed that Venetoclax (VEN), a BCL-2 inhibitor recently approved by the FDA for AML treatment, significantly decreases the intracellular level of methionine in AML cells. Based on these findings, we hypothesized that combining 8CA or 8AA with VEN can efficiently target the Methionine-MAT2A-S-adenosyl-methionine (SAM) axis in AML. Our results demonstrate that VEN and 8CA/8AA synergistically decrease the SAM biosynthesis and effectively target AML cells both in vivo and in vitro. These findings suggest the promising potential of combining 8CA/8AA and VEN for AML treatment by inhibiting Methionine-MAT2A-SAM axis and provide a strong rationale for our recently activated clinical trial.

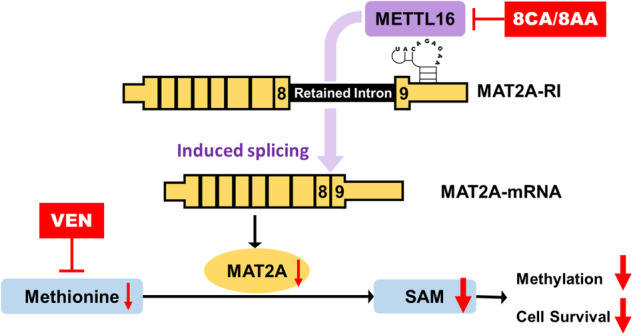

## Introduction

Acute myeloid leukemia (AML) is a hematological malignancy with a high prevalence in adults, significant recurrence rate and low survival [1, 2]. Historically the most effective treatment options were limited to intensive chemotherapy with subsequent allogeneic stem cell transplantation in fit individuals, but older patients and patients with comorbidities are often not candidates [3]. Recently, the selective BCL-2 inhibitor Venetoclax (VEN) has been approved by the FDA for AML treatment [4]. In AML, patients’ overall survival (OS) after VEN monotherapy was modest, but VEN in combination with the hypomethylating agents (HMA) azacitidine (AZA) or decitabine, or with low dose cytarabine (LDAC) dramatically increased OS of AML patients [5, 6]. However, about 30% of AML patients do not respond to these therapies and most patients eventually relapse, which is generally attributed to the drug-resistant leukemia stem cells (LSCs) [6, 7].

In contrast to normal hematopoietic stem cells, which depend on glycolysis for survival, LSCs specifically depend on oxidative phosphorylation driven by amino acid metabolism and/or fatty acid metabolism for their survival [8, 9]. The combination of VEN and AZA (VEN + AZA) globally inhibits the uptake of amino acids, thereby reducing the oxidative phosphorylation in de novo AML blasts [9]. However, relapsed LSCs upregulate fatty acid metabolism as a compensatory mechanism in response to the loss of amino acids [9]. Despite significant advances in AML therapy, outcomes remain poor, particularly in patients with relapsed/refractory (R/R) AML.

We have been investigating the potential of two RNA-directed nucleoside analogs, 8-chloro-adenosine (8CA) and 8-amino-adenosine (8AA), for AML treatment [10, 11]. We have conducted a phase 1 clinical trial of 8CA in R/R AML and witnessed significant clinical activity with all pretreated patients that their marrow leukemic blasts were sensitive to 8CA at nanomolar concentrations. Recognizing the fact that 8CA/8AA incorporate into RNA as chain terminators [12, 13], our lab integrated genetic and metabolic profiling to discover their effects on biological functions of AML. In our previous studies, we showed that 8CA, when used in combination with VEN, had synergistic effects on targeting oxidative phosphorylation through inhibiting fatty acid metabolism in both de novo and R/R primary AML blasts, with stronger efficacy than VEN + AZA [11]. Additionally, we observed that 8CA/8AA significantly inhibit the cysteine and methionine metabolism (C/M) pathway in AML.

C/M pathway is one of the most significantly altered metabolic pathways in LSCs in contrast to healthy blood cells, and cysteine and methionine have been validated as crucial dependencies of multiple cancers including AML [8, 14–19]. Methionine is the precursor for the synthesis of cysteine [20]. In the C/M pathway, methionine as an essential amino acid, is converted by the rate-limiting enzyme methionine-adenosyltransferase-2A (MAT2A) into S-adenosyl-methionine (SAM), which is the principal methyl-group donor for methylation reactions regulating gene expression [21]. To maintain SAM levels, the expression of MAT2A is regulated by the presence of the RNA methyltransferase-like 16 (METTL16) on MAT2A RNA [21]: When SAM levels are adequate, METTL16 methylates the hairpin structures of MAT2A RNA briefly, and then leaves it for degradation. When SAM levels are depleted, this methylation step is impaired, prolonging the occupancy of METTL16 on the hairpin structure, driving the splicing of MAT2A RNA and leading to the production of functional MAT2A protein for increased SAM biosynthesis. Additionally, the cleavage factor I_m_ (CFI_m_) 25 has been identified to mediate METTL16 regulated MAT2A splicing, and the process requires the arginine- and serine-rich regions (RS domains) of its binding partners CFI_m_68 and CFIm59.

Our preliminary data have revealed that 8CA/8AA decrease the expression of MAT2A, while VEN inhibits the uptake of methionine in AML cells. Thus, we hypothesize that 8CA/8AA can synergize with VEN to target the methionine-MAT2A-SAM axis in AML, and that 8CA/8AA plus VEN could represent a promising therapeutic strategy for AML treatment.

## Materials and methods

### Human samples

Human specimens were obtained from healthy individuals and individuals with AML registered at City of Hope National Medical Center, who consented to an Institutional Review Board (IRB)-approved protocol (IRB 14269). To isolate healthy peripheral blood mononuclear cells (PBMCs), fresh blood samples were processed using Ficoll-Paque Plus kit. To isolate leukemia stem cells (LSCs), bone marrow mononuclear cells (BMMCs) collected from R/R AML patients were stained with antibodies including Lineage (FITC-conjugated anti-CD2, anti-CD3, anti-CD4, anti-CD8, anti-CD14, anti-CD19, anti-CD20, anti-Mac-1, anti-CD56 and anti-CD235a), CD45 (BV510-conjugated anti-CD45), CD34 (PE-conjugated anti-CD34) and CD38 (BV605- or PE-Cy7-conjugated anti-CD38), and sorted through a BD FACSAria Fusion (BD Biosciences, San Jose, CA) for Lin^−^CD45^dim^CD34^+^CD38^-^ population.

### Animal study

The animal study was conducted under a protocol approved by the Institutional Animal Care and Use Committee at City of Hope (IACUC 22024). NOD/SCID/γ chain null (NSG) mice (female, 8–12 week old) were obtained from Jackson Laboratory (Sacramento, CA) and kept in micro-insulator cages in a pathogen-free condition. Mice were handled in laminar flow hoods. They were intravenously (i.v.) injected with 1 million AML blasts through tail veins. Five days after the injection, the mice were randomly divided into different treatment groups. 8CA (Tocris Bioscience, UK) (12.5 mg/kg/day, dissolved in PBS), 8AA (Santa Cruz Biotechnology, Dallas, TX) (5 mg/kg/day, dissolved in PBS) or vehicle control for 8CA/8AA was intraperitoneally (i.p.) administered. VEN (Selleckchem, Houston, TX) (20 mg/kg/day, dissolved in 10% ethanol, 60% Phosal 50 PG and 30% polyethylene glycol 400) or vehicle control for VEN was administered via oral gavage. The treatment lasted for 7 weeks. The bodyweight and temperature of the mice were measured once a week, and the survival was used as the endpoint measurement.

### Cell culture

The cell lines MV4-11 and KG-1a were obtained from the American Type Culture Collection (ATCC, Manassas, VA), and OCI-AML3 and Molm13 were obtained from DSMZ (Germany). They were maintained in Iscove’s Modified Dulbecco’s Medium (IMEM, Thermo Fisher Scientific, Irwindale, CA) containing 10% Fetal Bovine Serum (FBS, Thermo Fisher Scientific) and 100 U/mL penicillin/streptomycin at 37 °C with 5% CO_2_. Human cell lines purchased more than 6 months prior to submission of this manuscript and not frozen at an early passage were verified by ATCCs’ human short tandem repeat DNA profiling authentication service. The morphology of the cell lines was regularly monitored; potential mycoplasma contaminations were routinely monitored using a mycoplasma detection kit from Roche. All cell lines were mycoplasma-free.

Human LSCs were cultured using StemSpan serum-free expansion medium II (STEMCELL, Seattle, WA) supplemented with penicillin (100 U/mL) and streptomycin (100 mg/mL). Additionally, the medium was supplemented with stem cell factor (20 ng/mL), thrombopoietin (20 ng/mL), Flt3-L (20 ng/mL), IL-3 (10 ng/mL), and IL-6 (10 ng/mL).

### Cell viability

Cells were seeded in 96-well plates at a density of 2000 cells per well and then treated with 8CA/8AA/VEN or their combinations at a series of concentrations for 48 h. MTT assay was used for colorimetric measurement of proliferation of AML cell lines, following the manufacturer’s instructions (Promega, Madison, WI). CellTiter-Glo assay was used to measure metabolic activity of primary AML cells following the manufacturer’s instructions (Promega).

### Cell apoptosis

The Annexin-V and PI double-staining assay was used to determine cellular apoptosis. Briefly, cells were harvested and washed twice with Annexin-V binding buffer (BD Bioscience) and resuspended in 100 μL of the same buffer containing Annexin-V FITC and PI (BD Bioscience). The cells were then incubated in the dark at room temperature for 15 min, washed again and resuspended in 300 μL of buffer. The samples were then analyzed with an Accuri C6 flow cytometer (BD Bioscience).

### Colony formation assay

Cells were counted after treatment and seeded onto 24-well plates using Human Methylcellulose Complete Media (R&D Systems, Minneapolis, MN) according to the provided instructions. The cells were then cultured for a duration of 10 to 20 days, adjusting the duration based on the density of colonies observed. Then the plates were scanned using an Observer 7 microscope (Zeiss, White Plains, NY) and the colonies were counted using ImageJ software.

### AML patient dataset analysis

The Gene Data Set GDS1059 was obtained from NCBI GEO (Gene Expression Omnibus) and analyzed using the data analysis tools provided by NCBI Dataset Browser [22]. The dataset GS-DT-26 were acquired from GenomicScape (www.genomicscape.com) and analyzed using its survival analysis [23].

### Flow cytometry

At room temperature and in the dark, LSCs were first stained with the LIVE/DEAD™ kit (Thermo Fisher Scientific) for 20 min. Afterward, the cells were fixed and permeabilized using a Fixation and Cell Permeabilization kit (Thermo Fisher Scientific). Next, LSCs were collected and incubated with the primary antibodies anti-MAT2A (Abcam, Waltham, MA) or anti-METTL16 (Abcam) for 1 h, and then washed and incubated with the secondary antibody Alexa Fluor® 647 (Abcam) for 30 min. Finally, LSCs were washed for flow cytometry analysis.

Peripheral blood samples from mice were collected and the red blood cells (RBC) within each sample were lysed using RBC lysis buffer (Santa Cruz Biotechnology). The cells were then washed and stained with APC-conjugated anti-human CD45 antibody for flow cytometry analysis.

Spleen samples from mice were collected and stained with a LIVE/DEAD™ kit (Thermo Fisher Scientific) for 20 min, and fixed and permeabilized using a Fixation and Cell Permeabilization kit (Thermo Fisher Scientific). Then the spleen cells were incubated with the primary antibodies anti-H3K4me3 (Cell Signaling Technology, Danvers, Massachusetts) for 1 h, and then washed and incubated with the secondary antibody Alexa Fluor® 647 (Abcam) for 30 min. Finally, the spleen cells were washed for flow cytometry analysis.

Flow cytometry analysis was performed using Fortessa X-20 flow cytometer (BD Biosciences), and the acquired data were analyzed using FlowJo V10 (Tree Star, Ashland, OR).

### qRT-PCR

Total RNA was extracted from cells using Trizol (Invitrogen, Waltham, MA) and further purified with RNeasy kit (Qiagen, Germantown MD). Reverse transcriprion was performed using Omniscript RT Kit (Qiagen). Quantitative PCR reactions were run on a CFX96 Touch Real-Time PCR Detection System (Bio-Rad, Irvine, CA) using PowerUp™ SYBR™ Green Master Mix (Thermo Fisher Scientific). Primers were designed using Primer Bank and purchased from Integrated DNA Technologies. qRT-PCR analysis was conducted to measure the expression levels of the interested human genes (Supplementary Table 1).

### Western blotting

Cells were lysed using M-PER™ Mammalian Protein Extraction Reagent containing Halt™ Protease and Phosphatase Inhibitor Cocktail (Thermo Fisher Scientific). For histone extraction, the cells were lysed using Histone Extraction Kit (Abcam). The resulting products were mixed with loading buffer, heated at 95 °C for 5 min, and then cooled down to room temperature. The protein samples were then loaded onto SDS-PAGE gels (Bio-Rad) along with protein molecular weight markers (Bio-Rad) and electrophoresed at 100 V until the bands were separated sufficiently. The proteins were subsequently transferred to PVDF membranes using Trans-Blot Turbo Transfer System (Bio-Rad) for further analysis. The membranes were then blocked with blocking buffer (Li-Cor, Lincoln, NE) and incubated with primary antibodies at 4 °C overnight. Next, the membranes were washed with TBST and incubated with secondary antibodies at room temperature for 1 h, washed again and visualized using ChemiDoc Imaging System (Bio-Rad), or Odyssey Imager (Li-Cor).

### RNA immunoprecipitation (RIP)

The RIP-qPCR assay was conducted as previously reported [24]. Briefly, cells were crosslinked with 1% formaldehyde for 10 min and then the reaction was stopped by 0.25 M glycine for 5 min. The cells were subsequently washed by PBS twice and lysed by RIP buffer supplemented with Protease and Phosphatase Inhibitor Cocktail (Thermo Fisher Scientific), PMSF (Thermo Fisher Scientific), and RNase inhibitor (Invitrogen). After sonication for 10 cycles of 30 s on/30 s off at 4 °C using a Bioruptor Pico instrument (Diagenode, Denville, NJ), supernatant of cell lysate was harvested. 2 μg METTL16 antibody (IgG as control) was added to the supernatant. After incubation at 4 °C overnight, beads from Dynabeads Protein A Immunoprecipitation Kit (Invitrogen) were added and incubated for another 4 h. Beads were washed 3 times with RIP buffer. Finally, 100 µl elution buffer containing Proteinase K (Thermo Fisher Scientific) was used for further incubation at 42 °C for 1 h. Then the RNA can be extracted by Trizol or Phenol/Chloroform/Isoamyl Alcohol (Fisher BioReagents, Pittsburgh, PA) and then reverse transcribed to cDNA by using Omniscript RT Kit (Qiagen)according to manufacturer’s instructions. cDNA products with ~10-fold dilution were used as templates for qRT-PCR analysis.

### Statistical analysis

Potential synergistic or additive effects of drugs were determined using CompuSyn software (Cambridge, UK). Synergism, addition, and antagonism effects were defined by combination index values of <1.0, 1.0, and >1.0, respectively.

Data are representative of at least three independent experiments, unless otherwise stated. GraphPad Prism 8.3.0 was used to statistically analyze data, and data were shown as Mean ± SD. A two-tailed unpaired *t* test was used to compare two groups under the same condition, and a two-tailed paired test was used to compare two groups under the same series of conditions. Ordinary one-way ANOVA mixed with Tukey’s multiple comparisons test were used to compare multiple groups. Log-rank (Mantel–Cox) test was used to compare the survival curves between two groups. *P* < 0.05 was considered statistically significant. *P < 0.05, **P < 0.01, ***P < 0.001, and ****P < 0.0001.

## Results

### 8CA/8AA suppress the cysteine and methionine metabolism pathways in AML

**Fig. 1:**
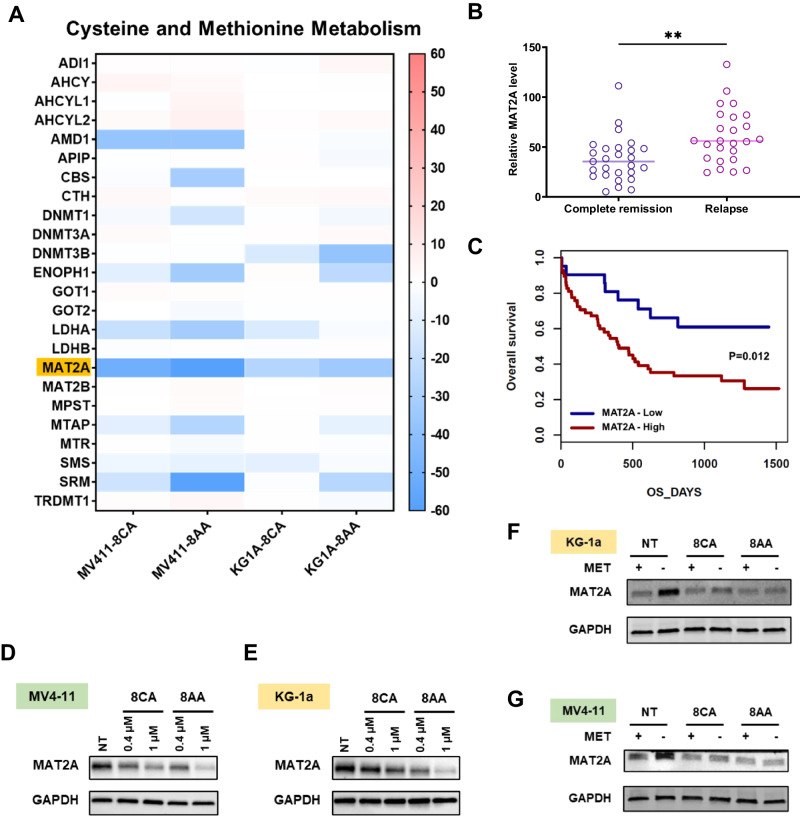
8CA/8AA downregulate MAT2A expression in the cysteine and methionine metabolism pathway. **A** AML cell lines MV4-11 and KG-1a were treated with 1 μM 8CA/8AA for 24 h, and the differentially expressed genes compared to nontreated group were analyzed by RNA sequencing and enriched using KEGG database and GSEA software (*n* = 2). The ranking metric scores of genes in the cysteine and methionine metabolism pathway are shown. **B**, **C** NCBI GEO and GenomicScape analysis of the expression level of MAT2A in AML patients. Complete remission (*n* = 27); relapse (n = 25); MAT2A-Low (*n* = 21, 26.6%), MAT2A-High (*n* = 58, 73.4%). **D**, **E** Protein levels of MAT2A in MV4-11 and KG-1a cells were measured through western blotting after 48 h treatment with vehicle (NT), 0.4 μM or 1 μM 8CA /8CA (*n* = 3). **F**, **G** Protein levels of MAT2A in MV4-11 and KG-1a cells were measured after 24 h pre-treatment with complete media or methionine-depleted media and followed by 24 h treatment with vehicle (NT) or 1 μM 8CA /8CA (*n* = 3). **p ≤ 0.01.

8CA/8AA have pan anti-cancer effects, and leukemias show the highest sensitivity to 8CA/8AA treatments in the NCI-60 human tumor cell line screening [25] (Supplementary Fig. 1A–D). We previously reported the anti-AML activity of 8CA at low nanomolar concentrations [10], and since 8AA shows promisingly lower IC50s than 8CA in multiple AML cell lines and has negligible effect on healthy blood cells as well as 8CA, we include both 8CA and 8AA in this study (Supplementary Fig. 1E–G). To identify their shared anti-AML mechanisms, we conducted RNA-seq analysis in two AML cell lines, MV4-11 and KG-1a, upon 8CA/8AA treatment. We screened the top ten downregulated/upregulated pathways and found that the cysteine and methionine metabolism (C/M) pathway is the only commonly downregulated pathway (Supplementary Fig. 2A–E). Additionally, one of the most downregulated genes in this pathway is MAT2A (Fig. 1A). Of note, there is a strong correlation between MAT2A expression levels and the prognosis of AML patients. Higher MAT2A levels are associated with higher relapse rates and worse overall survival rates (Fig. 1B, C).

### 8CA/8AA inhibit the expression of MAT2A in AML

**Fig. 2:**
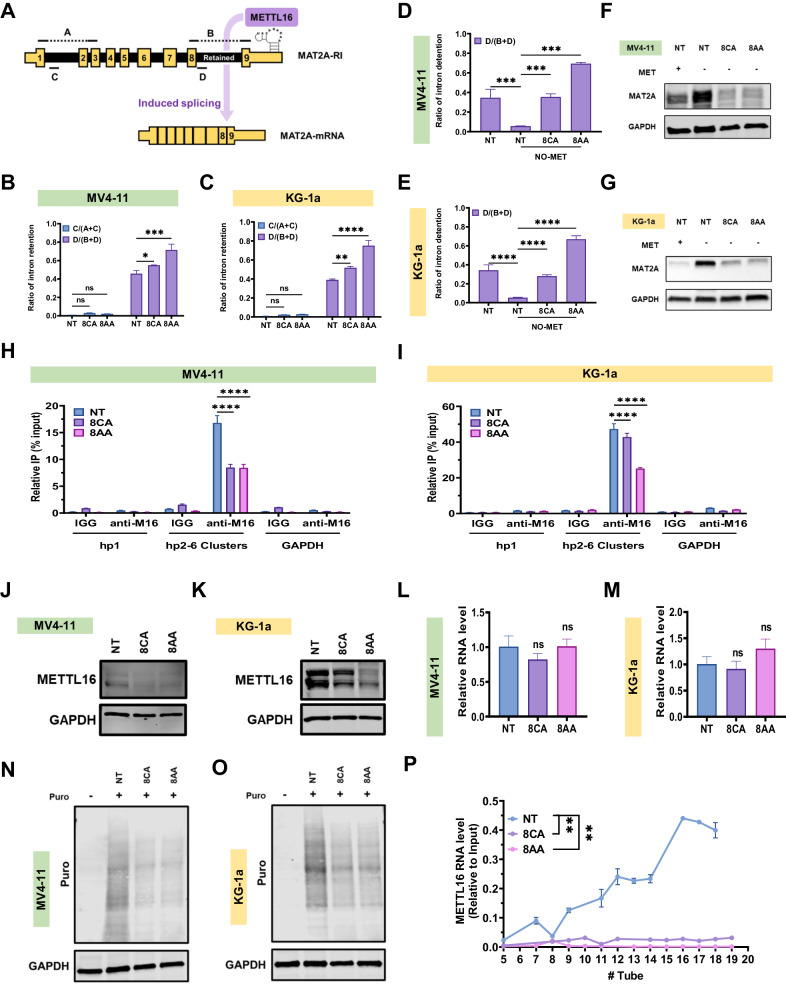
8CA/8AA increase intron retention in MAT2A RNA by decreasing METTL16 occupancy. **A** Diagram of primers covering different regions of MAT2A RNA. **B**, **C** The ratios of intron retention in MV4-11 and KG-1a cells were measured after 24 h treatment with vehicle (NT) or 1 μM 8CA/8AA (*n* = 3). **D**–**G** The ratios of intron retention in MV4-11 and KG-1a cells were measured after 24 h pre-treatment with complete media or methionine-depleted media and followed by 24 h treatment with vehicle (NT) or 1 μM 8CA/8AA (*n* = 3). The respective protein levels of MAT2A were measured as well. **H**, **I** The binding of hp1 and hp2–6 clusters of MAT2A RNA and GAPDH RNA to METTL16 protein in MV4-11 and KG-1a cells were measured after 48 h treatment with vehicle (NT) or 1 μM 8CA/8AA (*n* = 3). **J**–**M** Protein and RNA levels of METTL16 in MV4-11 and KG-1a cells were measured after 48 h treatment with vehicle (NT) or 1 μM 8CA/8AA (*n* = 3). **N**, **O** MV4-11 and KG-1a cells were pretreated with vehicle (NT) or 1 μM 8CA/8AA for 24 h, and then incubated with 10 μg/mL puromycin for 10 min. Puromycin incorporation during nascent protein synthesis was measured through western blotting. **P** The components of METTL16 RNA in MV4-11 cells after 24 h treatment with vehicle (NT) or 1 μM 8CA/8AA in different fractions extracted from the polysome profiling assay were measured by qRT-PCR and normalized to inputs (*n* = 3). ^ns^*p* > 0.05, *p ≤ 0.05, **p ≤ 0.01, ***p ≤ 0.001, ****p ≤ 0.0001.

At the protein level, MAT2A expression is inhibited by 8CA/8AA in a dose-dependent manner (Fig. 2D, E, Supplementary Fig. 1F, G). 8CA/8AA are chain terminators with global effects on gene expression, but interestingly other chain terminators do not downregulate the expression of MAT2A, suggesting inhibition of MAT2A expression is a unique mechanism of 8CA/8AA [10, 13, 26] (Supplementary Fig. 1H). MAT2A is the key rate-limiting enzyme in methionine metabolism, and MAT2A is overexpressed in a compensatory way when cells are starved from methionine [27]. However, the inhibitory effect of 8CA/8AA on MAT2A expression in both cell lines is still strong when methionine is depleted from the medium (Fig. 1F, G). Additionally, overexpression of MAT2A partially rescued the cell viability upon 8CA/8AA treatment (Supplementary Fig. 3A). Also, when AML cells are treated with 8CA/8AA, methionine depletion induces higher apoptosis and saturating cells with external SAM rescues the survival of AML cells, suggesting the antileukemic effect of 8CA/8AA could be enhanced when combined with methionine depletion, and 8CA/8AA could function on AML cells through impairing the synthesis of SAM by downregulating MAT2A (Supplementary Fig. 3D–G).

### 8CA/8AA inhibit the splicing of MAT2A RNA by impairing the synthesis of METTL16 protein

MAT2A RNA has two isoforms, and the successful splicing of the intron-retained isoform requires the occupancy of METTL16 protein on its hairpin structures [28]. To understand how 8CA/8AA affect the expression of MAT2A, we utilized the primers covering different regions A/B/C/D of the MAT2A transcript [29] (Fig. 2A). Under qRT-PCR conditions, A or B could only be detected when the respective intron covering C or D is spliced out [29]. Upon 8CA/8AA treatment, we observed a significant accumulation of the retained intron, which is represented by the increased detection ratio of D (Fig. 2B, C). However, splicing of the intron covering C is not affected by 8CA/8AA treatment, suggesting 8CA/8AA specifically impair the splicing of the retained intron, of which the splicing is regulated by the hairpin structure and METTL16 protein.

Since methionine depletion promotes the synthesis of MAT2A protein, we evaluated the level of the retained intron upon 8CA/8AA treatment in media containing no methionine (NO-MET). We observed that NO-MET treatment induces the splicing of the retained intron and that addition of 8CA/8AA severely impairs this induction (Fig. 2D, E), which is consistent with their paired MAT2A protein levels (Fig. 2F, G). Moreover, through RNA-immunoprecipitation we observed that 8CA/8AA reduce the binding of METTL16 RNA on the hairpin structure of MAT2A RNA (Fig. 2H, I).

**Fig. 3:**
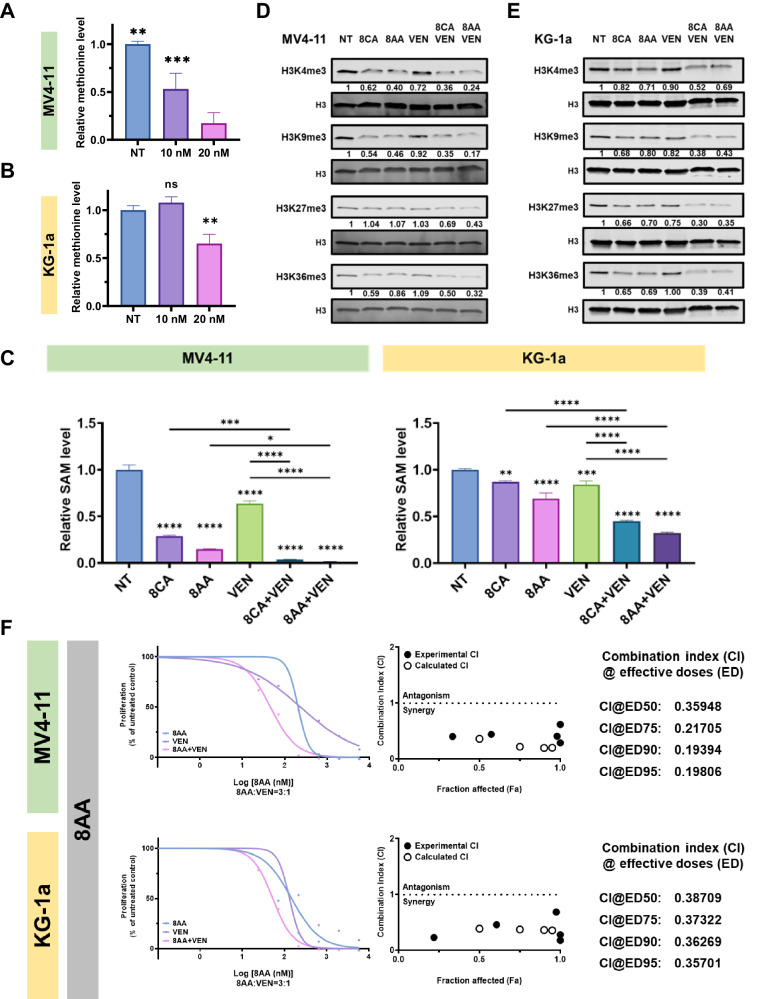
8CA/8AA and VEN synergistically target the viability and methylations of AML cell lines. **A**, **B** MV4-11 and KG-1a cells were treated with vehicle (NT), 10 nM or 20 nM VEN for 48 h, then the cellular methionine levels were measured through mass spectrometry (*n* = 3). **C**–**E** MV4-11 and KG-1a cells were treated with vehicle (NT), 1 μM 8CA/8AA, 0.02 μM VEN or their combinations for 48 h, then the intracellular SAM were measured through mass spectrometry (*n* = 3) and the histone methylations H3K4me3, H3k9me3, H3K27me3, and H3K36me3 were measured through western blotting. **F** MV4-11 and KG-1a cells were treated with 8AA, VEN or 8AA plus VEN at a series of concentrations for 48 h, and the cell viability was measured (*n* = 4). The combination index (CI) is calculated through CompuSyn software to define drug synergism (CI < 1.0), addition (CI = 1.0) and antagonism (CI > 1.0). *p ≤ 0.05, **p ≤ 0.01, ***p ≤ 0.001, ****p ≤ 0.0001.

We observed that other RNA methyltransferases METTL3/14 get upregulated at both RNA and protein levels upon 8CA/8AA treatment (Supplementary Fig. S4), but the expression of METTL16 at protein level is downregulated (Fig. 2J, K). And of note, 8CA/8AA doesn’t affect METTL16 expression at the RNA level (Fig. 2L, M). We hypothesized that 8CA/8AA interfere with either the synthesis or degradation of METTL16 protein in AML cells. Using cycloheximide (CHX) to stop nascent protein synthesis, we did not observe accelerated degradation of METTL16 upon treatment with 8CA/8AA (Supplementary Fig. 5A, B). Also, in a cell free assay system consisting of SAM and RNA oligos that can be methylated by METTL16, we did not observe any significant effect of 8CA/8AA on the enzymatic activity of METTL16 (Supplementary Fig. 5C–E). Consistently, the isothermal dose-response fingerprint (ITDRF) analysis suggested no direct binding between 8CA/8AA and METTL16 protein (Supplementary Fig. 5F). However, through the SUrface SEnsing of Translation assay, detecting the nascent incorporation of puromycin-labeled peptides (Fig. 2N, O) and ribosome profiling assay (Supplementary Fig. 6A, B), we found that 8CA/8AA affect the global translation speed and the translation speed of METTL16 RNA is strongly impaired as shown by the decreased polysome bindings compared to the control group [30] (Fig. 3P). In summary, our data suggest that 8CA/8AA impair the synthesis of METTL16 protein thereby inhibiting the splicing of MAT2A RNA.

### 8CA/8AA synergize with VEN in inhibiting SAM levels and histone methylations

We previously reported that 8CA synergize with Venetoclax (VEN) in targeting R/R AML, and our mass spec analysis revealed that VEN decreases cellular methionine levels in AML cells (Fig. 3A, B). Therefore, we hypothesized that 8CA/8AA synergize with VEN through inhibiting the Methionine-MAT2A-SAM axis. 8CA/8AA single agents decreased cellular SAM levels and the effect is more dramatic when used in combination with VEN (Fig. 3C). Moreover, we did not observe accumulation of S-adenosylhomocysteine (SAH), which is the byproduct when SAM is used for methylation reactions (Supplementary Fig. 7). This data suggests that the synergistically decreased SAM levels are caused by impaired synthesis, but not through consumption during methylations. Of importance, we observed synergistically and globally downregulated histone methylations upon 8CA/8AA plus VEN treatment (Fig. 3D, E). Also, we previously reported that 8CA synergizes with VEN in targeting the viability of AML cell lines [11], and we observed a similar synergistic antileukemia effect of 8AA and VEN (Fig. 3F).

Interestingly, we didn’t observe any changes in the methylations of DNA, total RNA, or mRNA (Supplementary Fig. 8). A possible reason could be that histone methyltransferases are more susceptible to changes in SAM levels caused by 8CA/8AA than other methyltransferases [31, 32]. Alternatively, it’s possibly caused by the compensatory upregulation of enzymes involved in these methylations upon 8CA/8AA treatment (Supplementary Fig. 4).

### 8CA/8AA synergize with VEN to target R/R AML blasts and leukemia stem cells (LSCs)

**Fig. 4:**
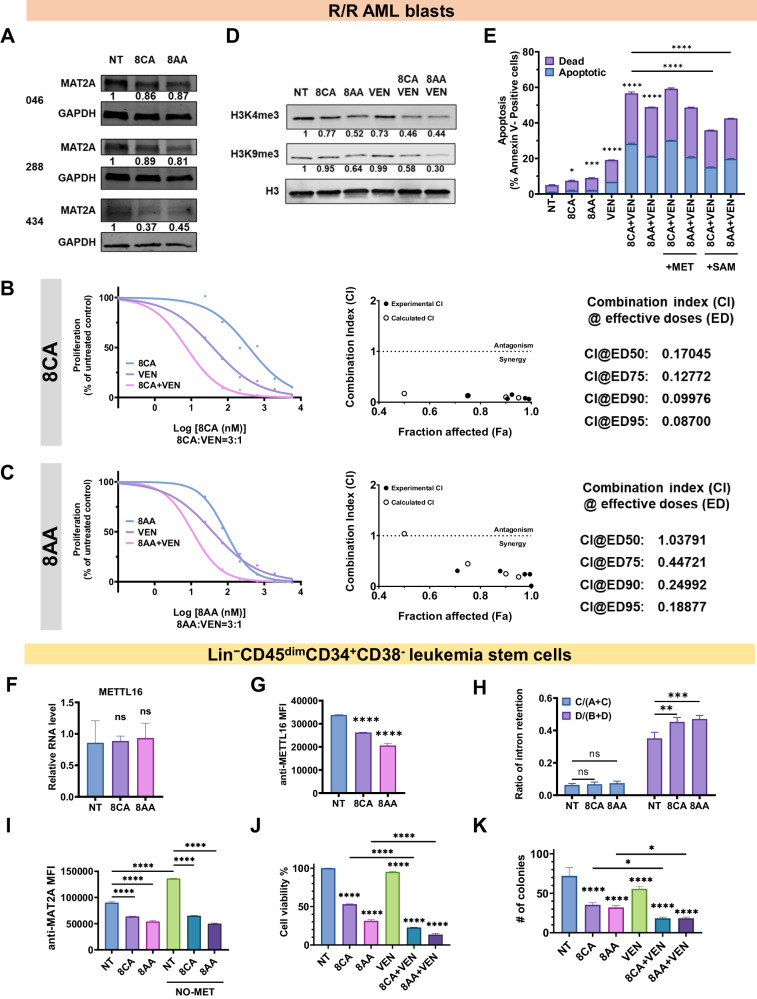
8CA/8AA and VEN synergistically target primary R/R AML blasts and Lin^−^CD45^dim^CD34^+^CD38^−^ LSCs. **A** Protein levels of MAT2A in primary R/R AML blasts isolated from three patients (#046, #288, #434) were measured after 48 h treatment with vehicle (NT) or 1 μM 8CA/8AA. **B**, **C** Primary R/R AML blasts were treated with 8CA/8AA, VEN or their combinations at a series of concentrations for 48 h, and the cell proliferations were measured (*n* = 4). The combination index (CI) is calculated through CompuSyn software to define drug synergism (CI < 1.0), addition (CI = 1.0) and antagonism (CI > 1.0). **D** Primary R/R AML blasts were treated with vehicle (NT), 1 μM 8CA/8AA, 0.02 μM VEN or their combinations for 48 h, then the histone methylations H3K4me3 and H3k9me3 were measured by western blotting (*n* = 3). **E** Cell apoptosis of primary R/R AML blasts was measured after 24 h pre-treatment with complete media or media containing 500 μM methionine ( + MET) or 500 μM SAM (+SAM), followed by 24 h treatment with vehicle (NT), 1 μM 8CA/8AA, 0.02 μM VEN or their combinations (*n* = 2). **F**, **G** RNA and protein levels of METTL16 in Lin^−^CD45^dim^CD34^+^CD38^-^ LSCs were measured after 48 h treatment with vehicle (NT) or 1 μM 8CA/8AA (*n* = 3). Median Fluorescence Intensity (MFI) of anti-METTL16 was measured through flow cytometry to represent the protein level of METTL16. **H** The ratios of intron retention in LSCs were measured after 24 h treatment with vehicle (NT) or 1 μM 8CA/8AA (*n* = 2). **I** Protein levels of MAT2A in LSCs (represented by MFI of anti-MAT2A) were measured after 24 h pre treatment with complete media or methionine-depleted media and followed by 24 h treatment with vehicle (NT) or 1 μM 8CA/8AA (*n* = 2). **J** Cell viability of LSCs was measured through flow cytometry after 24 h treatment with vehicle (NT), 1 μM 8CA/8AA, 0.02 μM VEN or their combinations (*n* = 2). **K** LSCs were pretreated with vehicle (NT), 1 μM 8CA/8AA, 0.02 μM VEN or their combinations for 24 h, and then cultured in 3D media at the same density to form colonies. Colonies were counted using microscopy and quantified (*n* = 3). ^ns^*p* > 0.05, *p ≤ 0.05, **p ≤ 0.01, ***p ≤ 0.001, ****p ≤ 0.0001.

We tested the effects of 8CA/8AA on primary AML blasts derived from various R/R AML patients and observed the consistent results that 8CA/8AA inhibit MAT2A expression and histone methylation levels and synergize with VEN to target the viability of AML blast cells (Fig. 4A–D).

**Fig. 5:**
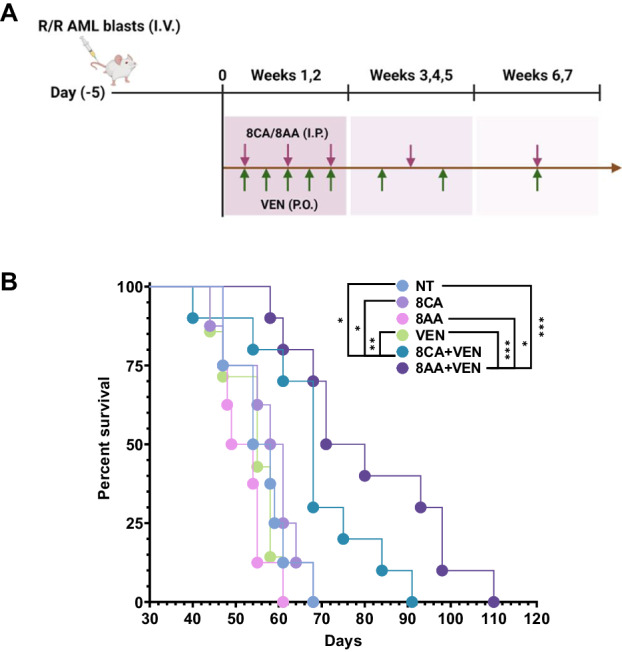
8CA/8AA and VEN synergistically inhibit the progression of R/R AML in vivo. **A** NSG mice were engrafted with 0.8 M R/R AML blasts, and after 5 days, they were treated with vehicle (*n* = 8), 12.5 mg/kg/day 8CA/5 mg/kg/day 8AA (*n* = 8), 20 mg/kg/day VEN (*n* = 7), or 8CA/8AA + VEN (*n* = 10) for 7 weeks. **B** The survival of mice receiving different treatments was monitored. *p ≤ 0.05, ***p ≤ 0.001.

To rescue cell viability upon 8CA/8AA treatment, we proposed to saturate AML blasts with external methionine and SAM. There had been debate on whether the cells could uptake external SAM from the cell culture medium [33]. Therefore, we measured the cellular level of methionine-cycle metabolites using mass spectrometry and observed that AML blasts have higher methionine levels compared to healthy blood cells and that adding SAM but not methionine could significantly upregulate the SAM levels in AML blasts when cultured in methionine-depleted medium (Supplementary Fig. 9). Adding external SAM but not methionine could partly overcome the induced apoptosis upon 8CA/8AA treatment in AML blasts, suggesting 8CA/8AA synergize with VEN in inhibiting the Methionine-MAT2A-SAM axis in AML blasts (Fig. 5E).

Since amino acid metabolism, especially methionine metabolism, is crucial for cancer stem cells to survive, we tested the effect of 8CA/8AA on Lin^−^CD45^dim^CD34^+^CD38^-^ LSCs [8, 15, 18, 34]. We consistently observed that 8CA/8AA affect METTL16 only at the protein but not the RNA level (Fig. 4F-G) with subsequent inhibition of MAT2A RNA splicing leading to decreased protein levels of MAT2A in LSCs (Fig. 4H, I). Moreover, 8CA/8AA synergize with VEN in inhibiting cell viability and colony formation ability of LSCs (Fig. 4J, K). These results suggest 8CA/8AA plus VEN synergize in targeting R/R AML blasts, as well as LSCs in vitro.

### 8CA and 8AA synergize with VEN in inhibiting the growth of R/R AML patient-derived blast cells in a murine PDX xenograft model

To evaluate the efficacy of 8CA/AA plus VEN in targeting R/R AML in vivo, we conducted an animal study. Immunodeficient NSG mice were engrafted with AML blasts derived from an R/R AML patient on Day (-5) (Fig. 5A, Supplementary Fig. 10A, B). Starting from Day 0, we initiated 7 weeks of treatment using vehicle (NT), single agents (8CA, 8AA, VEN), or their combinations (8CA/8AA + VEN). We observed that the single agents 8CA, 8AA, and VEN did not extend the survival of mice in the AML PDX model, compared to the control (NT) group (Fig. 5B). However, the combinations 8CA/8AA + VEN significantly prolonged survival while maintaining normal mice bodyweight and temperature (Fig. 5B, Supplementary Fig. 10C, D). Importantly, we observed significantly decreased histone methylation levels in the mononuclear spleen cells isolated from mice treated with the combinations 8CA/8AA + VEN (Supplementary Fig. 10E). These results suggest that 8CA/8AA synergize with VEN in targeting R/R AML in vivo.

## Discussion

8CA/8AA are known to share multiple common mechanisms of anti-cancer actions [10, 12, 13, 35]. In cells, 8CA/8AA are tri-phosphorylated into their cytotoxic metabolites 8-chloro-ATP and 8-amino-ATP, respectively. These metabolites have been demonstrated to affect RNA synthesis by incorporating into mRNA as chain terminators and to decrease endogenous ATP levels by blocking ATP synthase [12, 13, 36]. Treatment failures in AML are often attributed to the persistence of leukemia stem cells (LSCs). Cancer stem cells (CSCs) and AML cells are known to depend on methionine for their survival [8, 15, 16, 18, 37–40]. However, the impact of 8CA/8AA on amino acid metabolism, specifically methionine metabolism, has not been investigated before. We here report on the effects of 8AA/8CA on methionine metabolism in AML, as single agents and in combination with VEN.

Our study demonstrated 8CA/8AA as drugs that effectively inhibit MAT2A, which is the key rate-limiting enzyme in methionine metabolism converting methionine to S-adenosyl-methionine (SAM). Furthermore, we revealed the underlying mechanism behind their inhibitory action, showing that 8CA/8AA impact MAT2A splicing by influencing METTL16. The process may be mediated by CFI_m_ complex and requires future validation [41]. MAT2A is known to be overexpressed in many cancer types, particularly in leukemias [42]. In addition, MAT2A has been identified as a critical enzyme in CSCs, MLL-r leukemias, and cancers that lack methylthioadenosine-phosphorylase (MTAP), which accounts for 15% of all cancers [8, 18, 42, 43]. Based on our findings, 8CA/8AA demonstrate promising antileukemia potential by inhibiting MAT2A, making them favorable candidates to target MAT2A.

VEN is currently being utilized to treat AML patients in combination with HMAs or LDAC, most of which are DNA-directed compounds [3, 6, 7, 44]. Our findings underscore the innovative approach of combining VEN with the RNA-directed anti-cancer compounds 8CA/8AA (VEN + 8CA; VEN + 8AA) for AML treatment by targeting the methionine-MAT2A-SAM axis [10, 12, 13, 35]. Of note, we have recently completed a Phase 1 clinical trial (NCT02509546) at our institution using 8CA as single agent to determine the maximum tolerated dose and safety in relapsed/refractory (R/R) AML patients [45]. Results from this study showed promising albeit transient therapeutic activity. We have now initiated a first-in-human phase 1 clinical trial (NCT05263284) at our institution to investigate the 8CA/VEN combination in relapsed/refractory AML, aiming to determine whether this combination is safe and can achieve a more durable therapeutic response.

### Supplementary information

Supplementary Information

## Data Availability

The data generated in this study are available upon request from the corresponding author. The RNA-seq data have been deposited in the GEO repository. GSE247025.
