# Supplementary material for: 8-Cl-Ado and 8-NH2-Ado synergize with venetoclax to target the methionine-MAT2A-SAM axis in acute myeloid leukemia
Source: Leukemia. 2024 Apr 20;38(6):1236–45. doi: 10.1038/s41375-024-02222-w (PMC11147765; doi:10.1038/s41375-024-02222-w)
Supplement: Supplementary file 1 — Supplementary Information [file 41375_2024_2222_MOESM1_ESM.pdf]

Supplementary information

Supplementary Table 1. Primers used for qRT-PCR.

**METTL16**	**F 5'→3'**	CTCTGACGTGTACTCTCCTAAGG
	**R 5'→3'**	TACCAGCCATTCAAGGTTGCT
**METTL3**	**F 5'→3'**	TTGTCTCCA ACCTTCCGTAGT
	**R 5'→3'**	CCAGATCAGAGAGGTGGTGTAG
**METTL14**	**F 5'→3'**	AGTGCCGACAGCATTGGTG
	**R 5'→3'**	GGAGCAGAGGTATCATAGGAAGC
**MAT2A hp1**	**F 5'→3'**	CATGGGAAGTGCCCAAAAAG
	**R 5'→3'**	CAGAGCTTGAAGGCTTCTCT
**MAT2A hp2-6**	**F 5'→3'**	ATTCTGGGGTATGGCGTAAG
	**R 5'→3'**	TAAAAGCTGCCATCTGAGGT
**MAT2A A**	**F 5'→3'**	CCACCCAGATAAGATTTGTGACC
	**R 5'→3'**	GATGTAATTTCCCCAGCAAGAAG
**MAT2A B**	**F 5'→3'**	TCAGAAGAGTGAGAGAGAGCTAT
	**R 5'→3'**	CCATAGGCTGCAGTCCTC
**MAT2A C**	**F 5'→3'**	TTTCTGCCCTTAGGATGACC
	**R 5'→3'**	CAAGATCCTGGGTTTGTCCT
**MAT2A D**	**F 5'→3'**	AAGTGGGTTGCTCAAGGTTT
	**R 5'→3'**	CCTGGCTCAACAAATACGAA
**WTAP**	**F 5'→3'**	CTTCCCAAGAAGGTTCGATTGA
	**R 5'→3'**	TCAGACTCTCTTAGGCCAGTTAC
**FTO**	**F 5'→3'**	ACTTGGCTCCCTTATCTGACC
	**R 5'→3'**	TGTGCAGTGTGAGAAAGGCTT
**ALKBH5**	**F 5'→3'**	CGGCGAAGGCTACACTTACG
	**R 5'→3'**	CCACCAGCTTTTGGATCACCA
**GADPH**	**F 5'→3'**	GGAGCGAGATCCCTCCAAAAT
	**R 5'→3'**	GGCTGTTGTCATACTTCTCATGG

S1

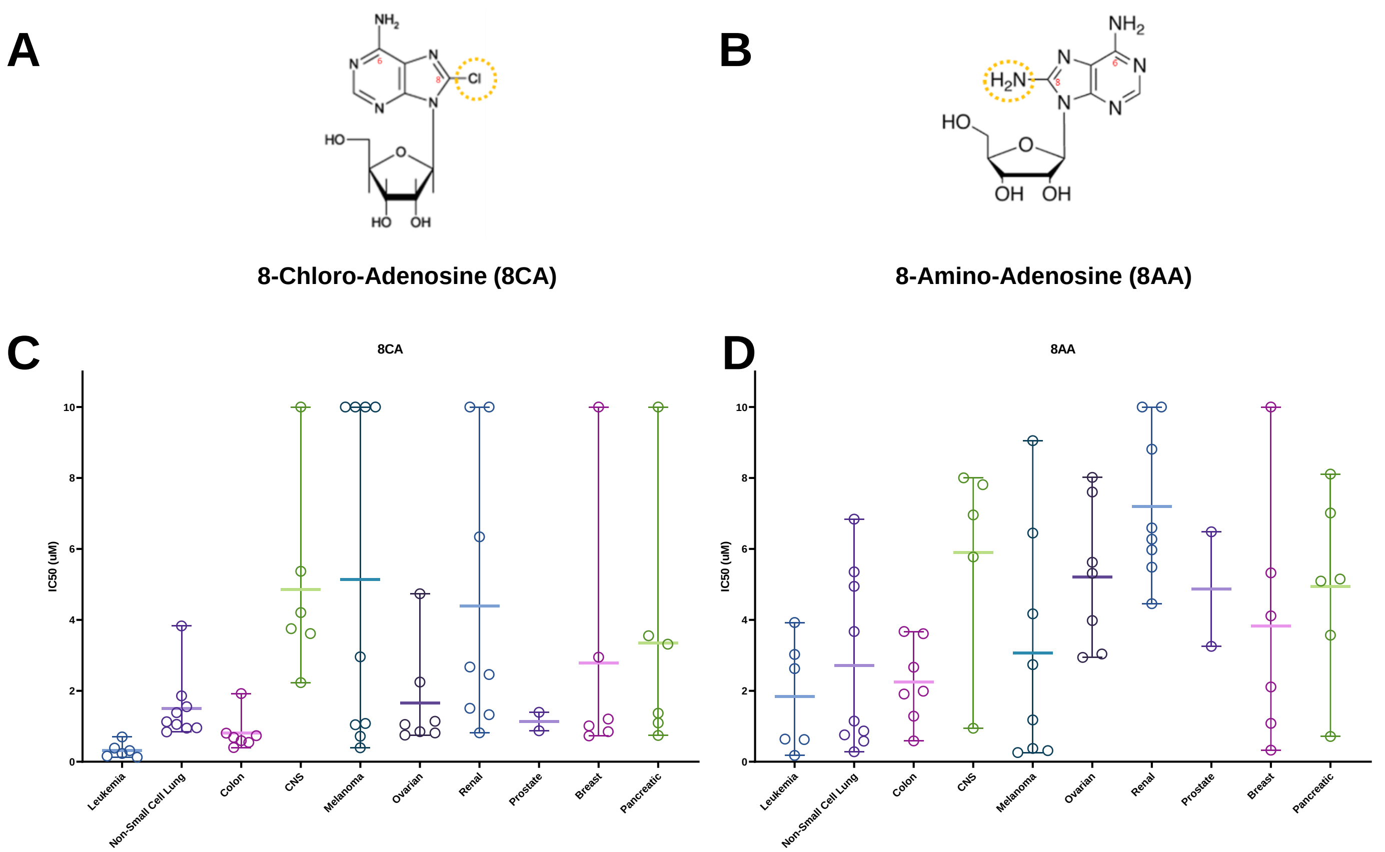

E

AML Cell Line	OCI-AML3		MOLM13		MV4-11		KG-1a	
Compound	8CA	8AA	8CA	8AA	8CA	8AA	8CA	8AA
48h IC50 (nM)	362.3	292.7	309.8	176.9	392.1	194.4	384.7	323.2

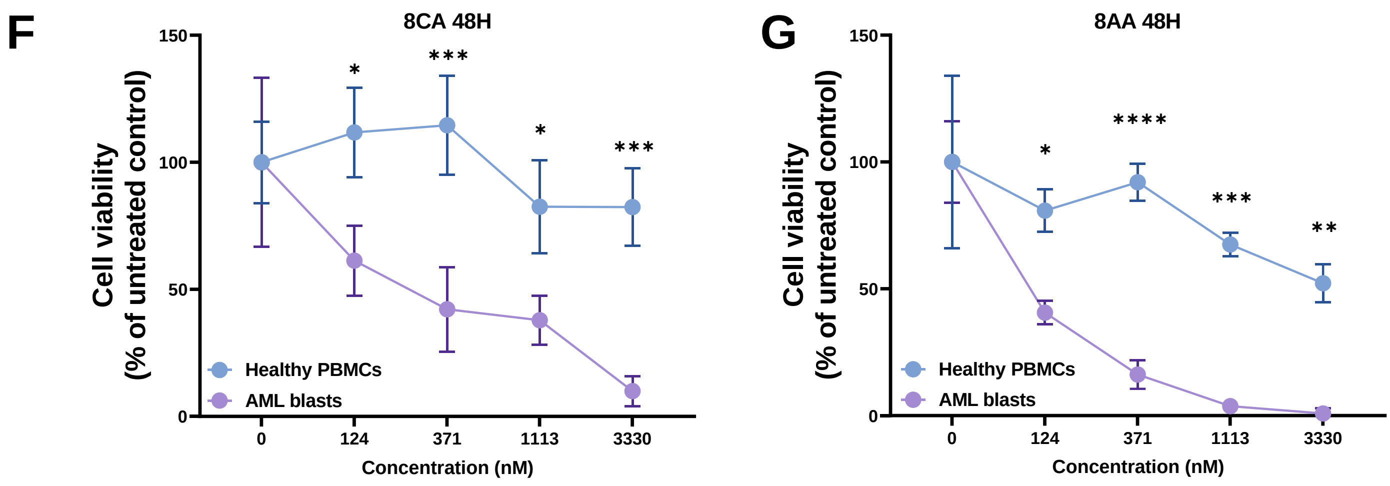

Supplementary Figure 1. 8CA/8AA exhibit favorable antileukemic activities. A-B Structures of 8-chloro-adenosine (8CA) and 8-amino-adenosine (8AA). **C-D** IC50s of 8CA/8AA in different cancer cell lines (NCI-60 Screen). **E** Four AML cell lines were treated with 8CA/8AA in a series of concentrations for 48 h and the cell viabilities were measured. IC50s were determined by "Absolute IC50, X represents log(concentration)" equation, measured by GraphPad Prism software (n=4). **F-G** PBMCs isolated from healthy donor and primary blasts isolated from the bone marrow of AML patients were treated with 8CA/8AA in a series of concentrations and the cell viability was measured (n=3). $^{ns}p > 0.05$, $^{*}p \leq 0.05$, $^{**}p \leq 0.01$, $^{***}p \leq 0.001$, $^{****}p \leq 0.0001$.

S2

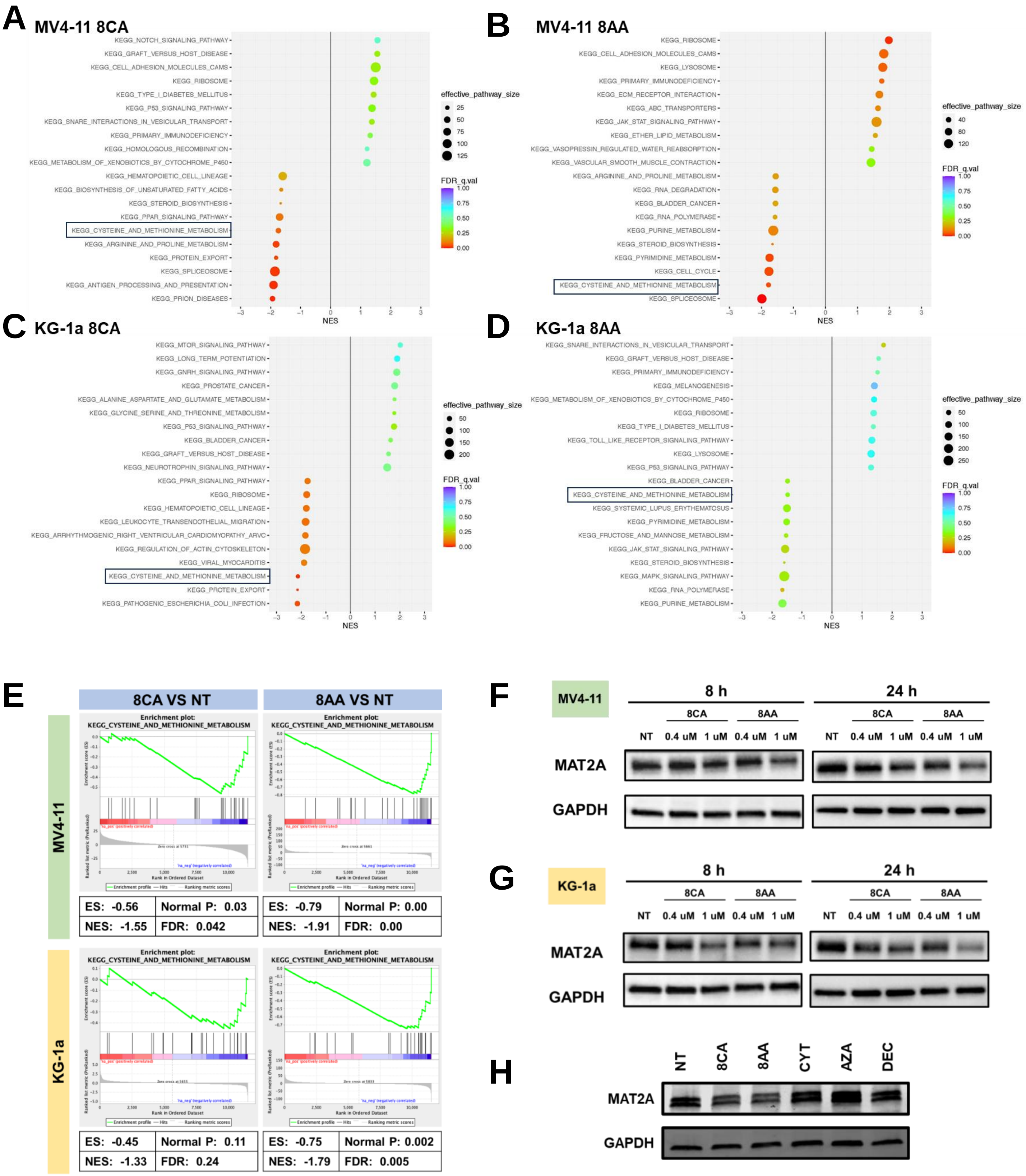
A
MV4-11 8CA
KEGG_NOTCH_SIGNALING_PATHWAY
KEGG_GRAFT_VERSUS_HOST_DISEASE
KEGG_CELL_ADHESION_MOLECULES_CAMS
KEGG_RIBOSOME
KEGG_TYPE_I_DIABETES_MELLITUS
KEGG_P53_SIGNALING_PATHWAY
KEGG_SNARE_INTERACTIONS_IN_VESICULAR_TRANSPORT
KEGG_PRIMARY_IMMUNODEFICIENCY
KEGG_HOMOLOGOUS_RECOMBINATION
KEGG_METABOLISM_OF_XENOBIOTICS_BY_CYTOCHROME_P450
KEGG_HEMATOPOIETIC_CELL_LINEAGE
KEGG_BIOSYNTHESIS_OF_UNSATURATED_FATTY_ACIDS
KEGG_STEROID_BIOSYNTHESIS
KEGG_PPAR_SIGNALING_PATHWAY
KEGG_CYSTEINE_AND_METHIONINE_METABOLISM
KEGG_ARGININE_AND_PROLINE_METABOLISM
KEGG_PROTEIN_EXPORT
KEGG_SPLICEOSOME
KEGG_ANTIGEN_PROCESSING_AND_PRESENTATION
KEGG_PRION_DISEASES
NES
effective_pathway_size
FDR_q.val
B
MV4-11 8AA
KEGG_RIBOSOME
KEGG_CELL_ADHESION_MOLECULES_CAMS
KEGG_LYSOSOME
KEGG_PRIMARY_IMMUNODEFICIENCY
KEGG_ECM_RECEPTOR_INTERACTION
KEGG_ABC_TRANSPORTERS
KEGG_JAK_STAT_SIGNALING_PATHWAY
KEGG_ETHER_LIPID_METABOLISM
KEGG_VASOPRESSIN_REGULATED_WATER_REABSORPTION
KEGG_VASCULAR_SMOOTH_MUSCLE_CONTRACTION
KEGG_ARGININE_AND_PROLINE_METABOLISM
KEGG_RNA_DEGRADATION
KEGG_BLADDER_CANCER
KEGG_RNA_POLYMERASE
KEGG_PURINE_METABOLISM
KEGG_STEROID_BIOSYNTHESIS
KEGG_PYRIMIDINE_METABOLISM
KEGG_CELL_CYCLE
KEGG_CYSTEINE_AND_METHIONINE_METABOLISM
KEGG_SPLICEOSOME
NES
effective_pathway_size
FDR_q.val
C
KG-1a 8CA
KEGG_MTOR_SIGNALING_PATHWAY
KEGG_LONG_TERM_POTENTIATION
KEGG_GNRH_SIGNALING_PATHWAY
KEGG_PROSTATE_CANCER
KEGG_ALANINE_ASPARTATE_AND_GLUTAMATE_METABOLISM
KEGG_GLYCINE_SERINE_AND_THREONINE_METABOLISM
KEGG_P53_SIGNALING_PATHWAY
KEGG_BLADDER_CANCER
KEGG_GRAFT_VERSUS_HOST_DISEASE
KEGG_NEUROTROPHIN_SIGNALING_PATHWAY
KEGG_PPAR_SIGNALING_PATHWAY
KEGG_RIBOSOME
KEGG_HEMATOPOIETIC_CELL_LINEAGE
KEGG_LEUKOCYTE_TRANSENDOTHELIAL_MIGRATION
KEGG_ARRHYTHMOGENIC_RIGHT_VENTRICULAR_CARDIOMYOPATHY_ARVC
KEGG_REGULATION_OF_ACTIN_CYTOSKELETON
KEGG_VIRAL_MYOCARDITIS
KEGG_CYSTEINE_AND_METHIONINE_METABOLISM
KEGG_PROTEIN_EXPORT
KEGG_PATHOGENIC_ESCHERICHIA_COLI_INFECTION
NES
effective_pathway_size
FDR_q.val
D
KG-1a 8AA
KEGG_SNARE_INTERACTIONS_IN_VESICULAR_TRANSPORT
KEGG_GRAFT_VERSUS_HOST_DISEASE
KEGG_PRIMARY_IMMUNODEFICIENCY
KEGG_MELANOGENESIS
KEGG_METABOLISM_OF_XENOBIOTICS_BY_CYTOCHROME_P450
KEGG_RIBOSOME
KEGG_TYPE_I_DIABETES_MELLITUS
KEGG_TOLL_LIKE_RECEPTOR_SIGNALING_PATHWAY
KEGG_LYSOSOME
KEGG_P53_SIGNALING_PATHWAY
KEGG_BLADDER_CANCER
KEGG_CYSTEINE_AND_METHIONINE_METABOLISM
KEGG_SYSTEMIC_LUPUS_ERYTHEMATOSUS
KEGG_PYRIMIDINE_METABOLISM
KEGG_FRUCTOSE_AND_MANNOSE_METABOLISM
KEGG_JAK_STAT_SIGNALING_PATHWAY
KEGG_STEROID_BIOSYNTHESIS
KEGG_MAPK_SIGNALING_PATHWAY
KEGG_RNA_POLYMERASE
KEGG_PURINE_METABOLISM
NES
effective_pathway_size
FDR_q.val
E
8CA VS NT
8AA VS NT
MV4-11
Enrichment plot: KEGG_CYSTEINE_AND_METHIONINE_METABOLISM
ES: -0.56 Normal P: 0.03
NES: -1.55 FDR: 0.042
ES: -0.79 Normal P: 0.00
NES: -1.91 FDR: 0.00
KG-1a
ES: -0.45 Normal P: 0.11
NES: -1.33 FDR: 0.24
ES: -0.75 Normal P: 0.002
NES: -1.79 FDR: 0.005
F
MV4-11
8 h
24 h
8CA 8AA
NT 0.4 uM 1 uM 0.4 uM 1 uM
MAT2A
GAPDH
G
KG-1a
8 h
24 h
8CA 8AA
NT 0.4 uM 1 uM 0.4 uM 1 uM
MAT2A
GAPDH
H
NT 8CA 8AA CYT AZA DEC
MAT2A
GAPDH

Supplementary Figure 2. 8CA/8AA downregulate the cysteine and methionine metabolism pathway. **A-E** AML cell lines MV4-11 and KG-1a were treated with 1 μM 8CA/8AA for 8 h, and the differentially expressed genes compared to nontreated group were analyzed by RNA sequencing and enriched using KEGG database and GSEA software (n=2). **F-G** Protein levels of MAT2A in MV4-11 and KG-1a were measured after 8 h and 24 h treatment with vehicle (NT), 0.4 μM or 1 μM 8CA/8AA. **H** Protein levels of MAT2A in MV4-11 cells were measured after 48 h treatment with vehicle (NT), 1 μM 8CA, 8AA, cytarabine (CYT), azacitidine (AZA) or decitabine (DEC).

S3

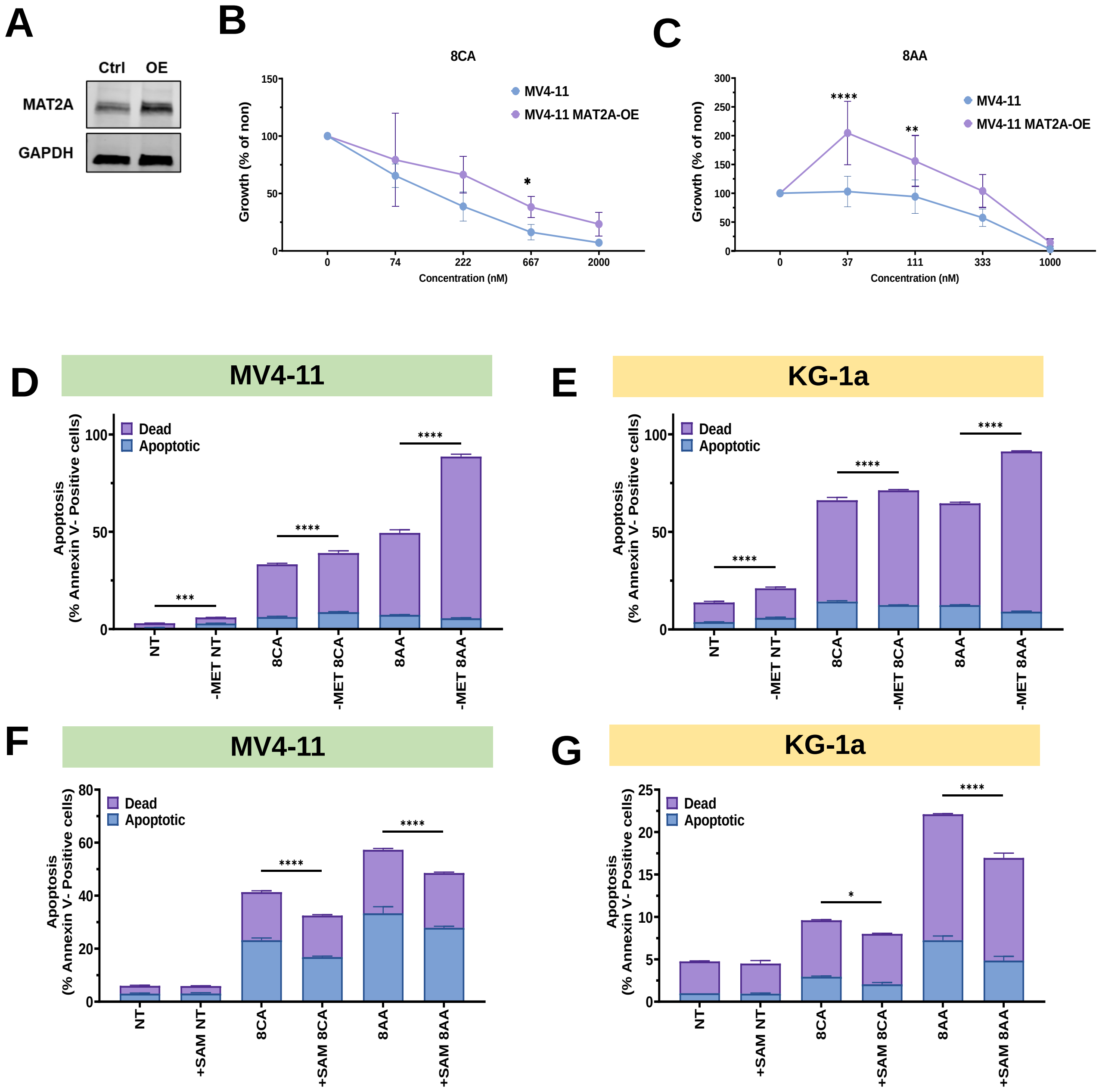
A
Ctrl
OE
MAT2A
GAPDH
B
8CA
Growth (% of non)
MV4-11
MV4-11 MAT2A-OE
Concentration (nM)
C
8AA
D
MV4-11
Dead
Apoptotic
Apoptosis
(% Annexin V- Positive cells)
NT
-MET NT
8CA
-MET 8CA
8AA
-MET 8AA
E
KG-1a
F
MV4-11
+SAM NT
+SAM 8CA
+SAM 8AA
G
KG-1a

Supplementary Figure 3. 8CA/8AA function on AML through inhibiting MAT2A. A-C Protein levels of MAT2A in control-treated MV4-11 cells (Ctrl) and MV4-11 cells overexpressing (OE) MAT2A were measured to confirm transduction efficiency. Cell viability of the two cell lines after 24 h treatment with 8CA/8AA in a series of concentrations was measured. **D-E** Cell apoptosis of MV4-11 and KG-1a was measured through flow cytometry after 24 h treatment with vehicle (NT) or 1 μM 8CA/8AA in complete media or methionine-depleted media (n=3). **F-G** Cell apoptosis of MV4-11 and KG-1a was measured after 24 h pre-treatment with complete media or media containing 500 μM SAM and followed by 24 h treatment with vehicle (NT) or 1 μM 8CA/8AA (n=2). $^{ns}p > 0.05$, $^{*}p \leq 0.05$, $^{**}p \leq 0.01$, $^{***}p \leq 0.001$, $^{****}p \leq 0.0001$.

S4

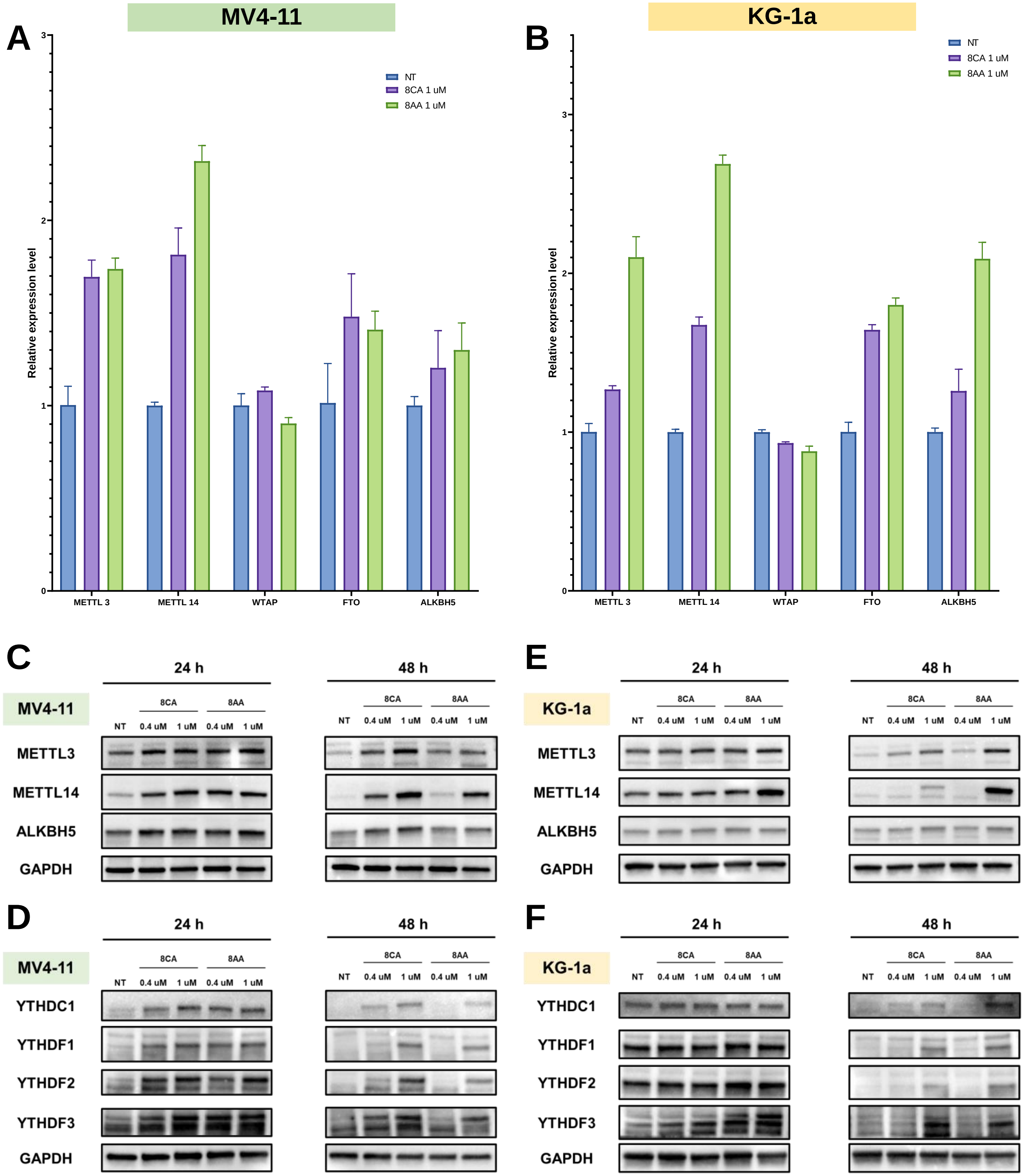

MV4-11
KG-1a
A
B
Relative expression level
NT
8CA 1 uM
8AA 1 uM
0
1
2
3
METTL 3
METTL 14
WTAP
FTO
ALKBH5
C
D
E
F
24 h
48 h
8CA
8AA
NT
0.4 uM
1 uM
METTL3
METTL14
ALKBH5
GAPDH
YTHDC1
YTHDF1
YTHDF2
YTHDF3

Supplementary Figure 4. 8CA/8AA upregulate the expression levels of RNA methylation related enzymes. A-B RNA levels of genes of interest in MV4-11 and KG-1a were measured after 24 h treatment with vehicle (NT) or 1 μM 8CA/8AA (n=3). The RNA levels are normalized to GAPDH expression. **C-F** Protein levels of genes of interest in MV4-11 and KG-1a were measured after 24 h or 48 h treatment with vehicle (NT), 0.4 μM or 1 μM 8CA/8AA.

S5

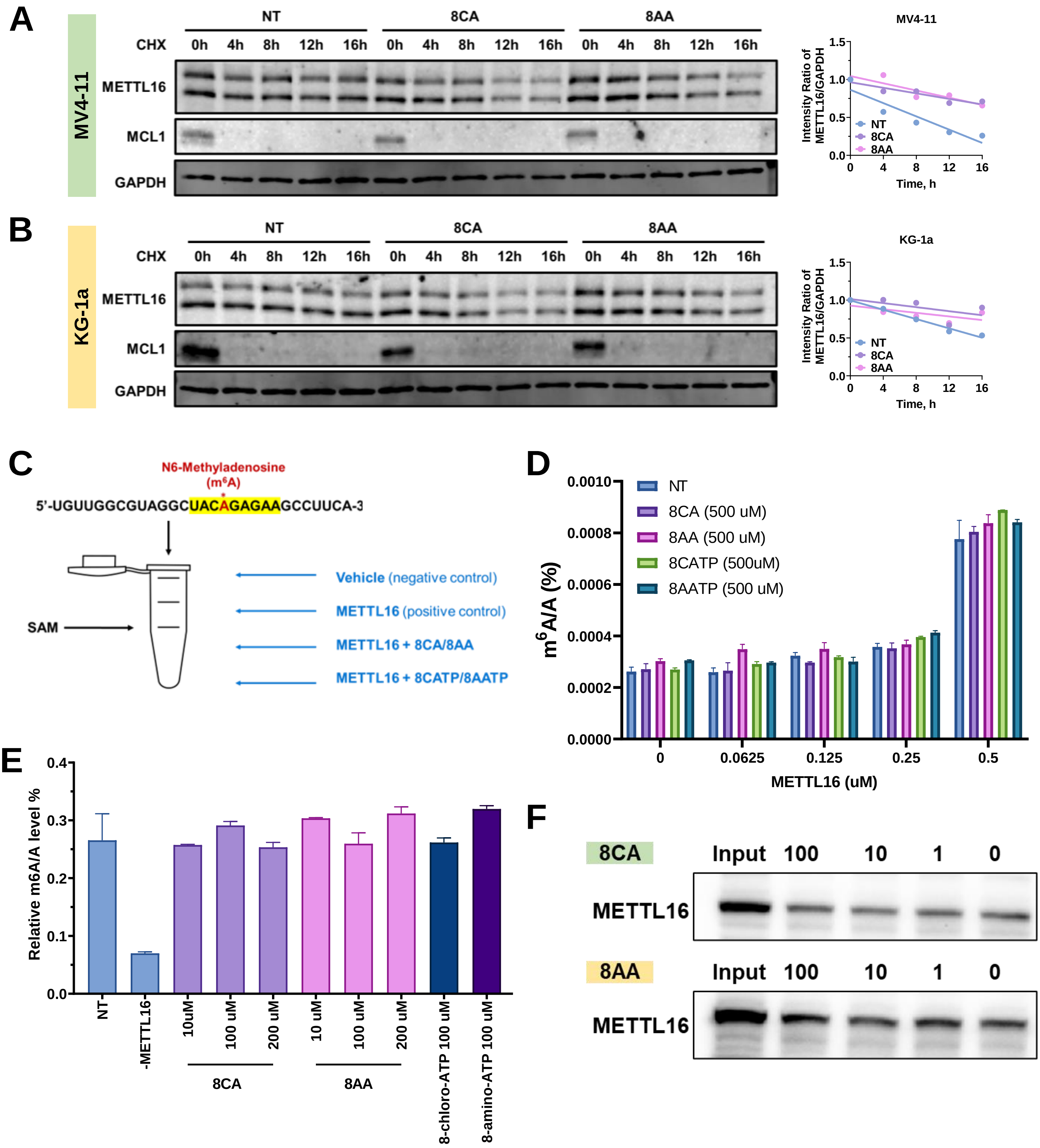
A
MV4-11
NT
8CA
8AA
CHX
0h
4h
8h
12h
16h
METTL16
MCL1
GAPDH
MV4-11
Intensity Ratio of METTL16/GAPDH
Time, h
NT
8CA
8AA
B
KG-1a
NT
8CA
8AA
CHX
0h
4h
8h
12h
16h
METTL16
MCL1
GAPDH
KG-1a
Intensity Ratio of METTL16/GAPDH
Time, h
C
N6-Methyladenosine
(m6A)
5'-UGUUGGCGUAGGCUACAGAGAAGCCUUCA-3
SAM
Vehicle (negative control)
METTL16 (positive control)
METTL16 + 8CA/8AA
METTL16 + 8CATP/8AATP
D
NT
8CA (500 uM)
8AA (500 uM)
8CATP (500uM)
8AATP (500 uM)
m6A/A (%)
METTL16 (uM)
E
Relative m6A/A level %
NT
-METTL16
10uM
100 uM
200 uM
10 uM
100 uM
200 uM
8CA
8AA
8-chloro-ATP 100 uM
8-amino-ATP 100 uM
F
8CA
Input
100
10
1
0
METTL16
8AA
Input
100
10
1
0
METTL16

Supplementary Figure 5. 8CA/8AA do not affect the degradation or enzymatic activity of METTL16 protein. A-B MV4-11 and KG-1a cells were pretreated with 1 μM 8CA/8AA for 12 h and the protein levels of METTL16 were measured at multiple time points through Western blot after the protein synthesis was stopped by adding 100 μg/mL cycloheximide (CHX). **C-E** RNA oligos containing the sequence that can be methylated by METTL16 were incubated with SAM, and then incubated with vehicle (NT), METTL16 protein, or METTL16 plus 8CA/8AA or their cellular metabolites 8CATP/8AATP at increasing concentrations. The methylation level represented by m6A/A ratio was measured through QQQ-mass spectrometry. **F** ITDRF detection of interaction between 8CA/8AA and METTL16 protein.

S6

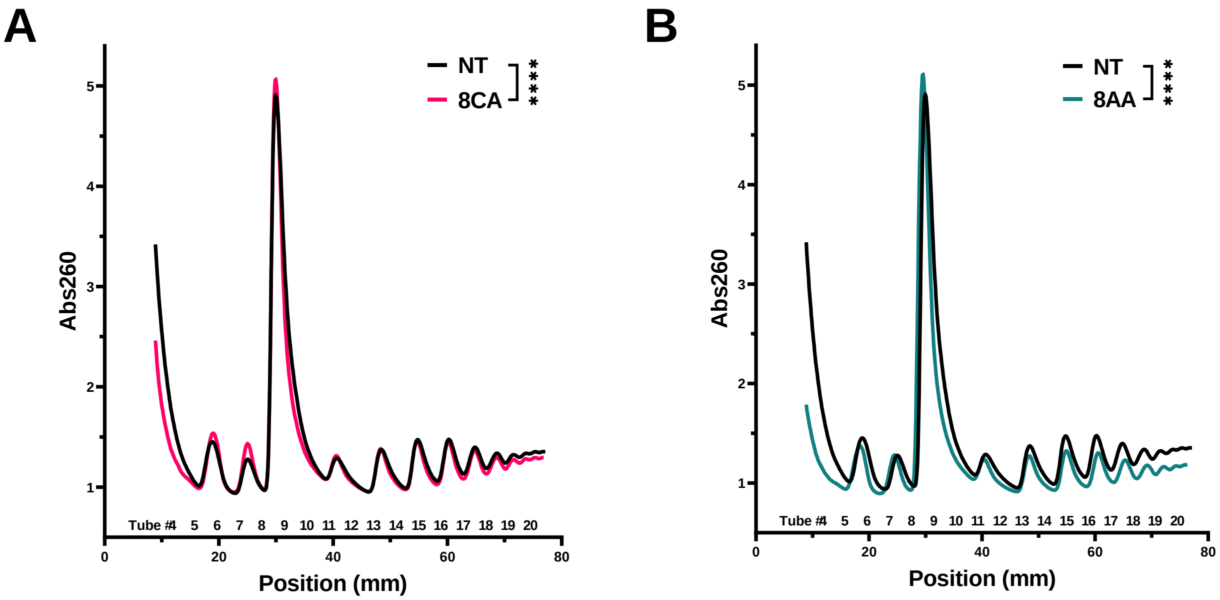

Supplementary Figure 6. Polysome profiles. A-B Polysome profiles of MV4-11 after 24 h treatment with vehicle (NT) or 1 μM 8CA/8AA were determined by sucrose density gradient ultracentrifugation (n=3). ****p≤0.0001.

S7

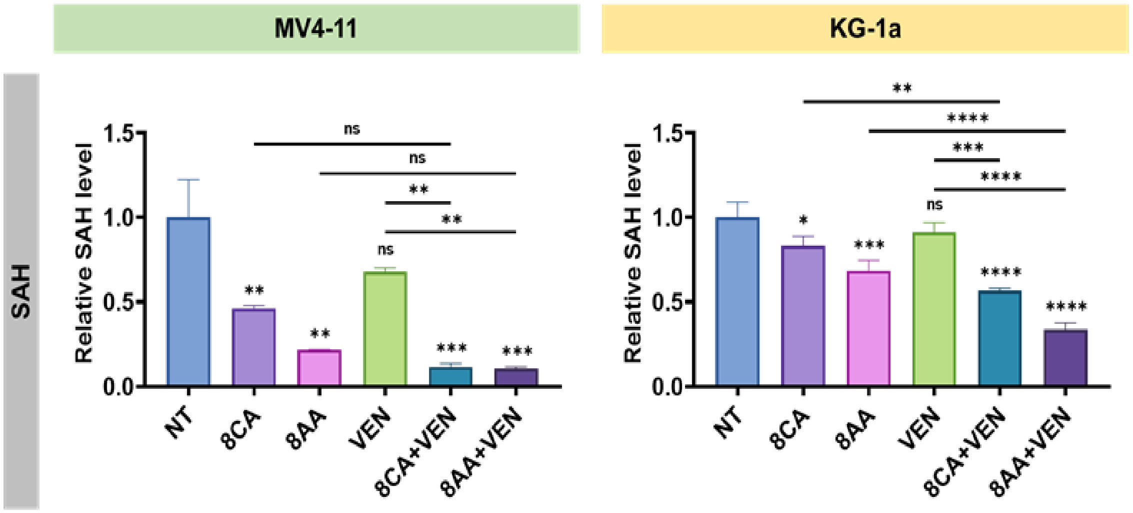

Supplementary Figure 7. 8CA/8AA do not increase SAH levels in AML cell lines. MV4-11 and KG-1a cells were treated with vehicle (NT), 1 μM 8CA/8AA, 0.02 μM VEN or their combinations for 48 h, then the intracellular SAH levels were measured through mass spectrometry (n=3). $^{ns}p > 0.05$, *$p \leq 0.05$, **$p \leq 0.01$, ***$p \leq 0.001$, ****$p \leq 0.0001$.

S8

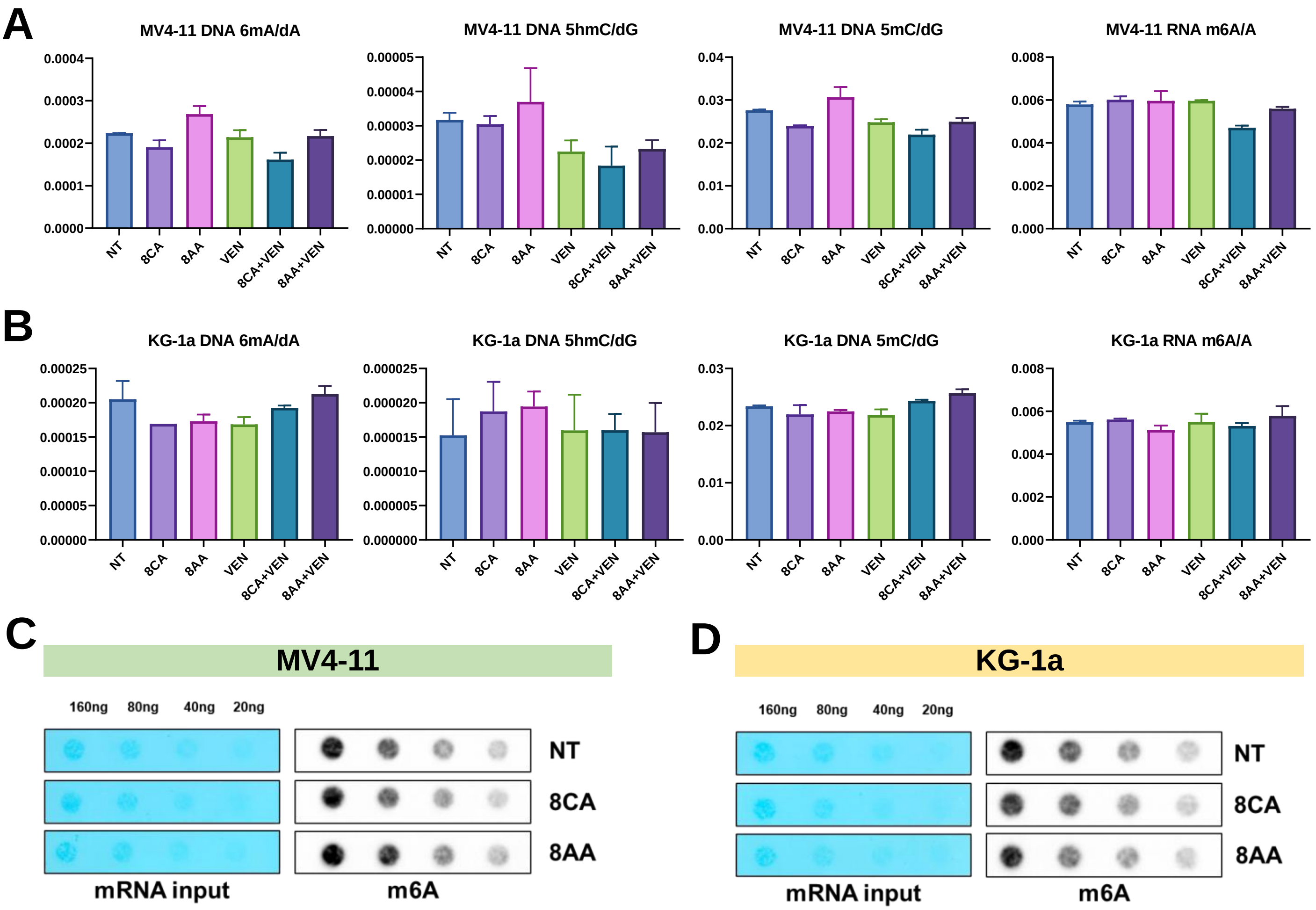

Supplementary Figure 8. 8CA/8AA do not affect DNA or RNA methylations. A-B Methylation levels of total DNA and total RNA in MV4-11 and KG-1a were measured through mass spectrometry after 48 h treatment with vehicle (NT), 1 μM 8CA/8AA, 0.02 μM VEN or their combinations. **C-D** Methylation levels of mRNA in MV4-11 and KG-1a were measured through dot blot assay after 48 h treatment with vehicle (NT) or 1 μM 8CA/8AA.

S9

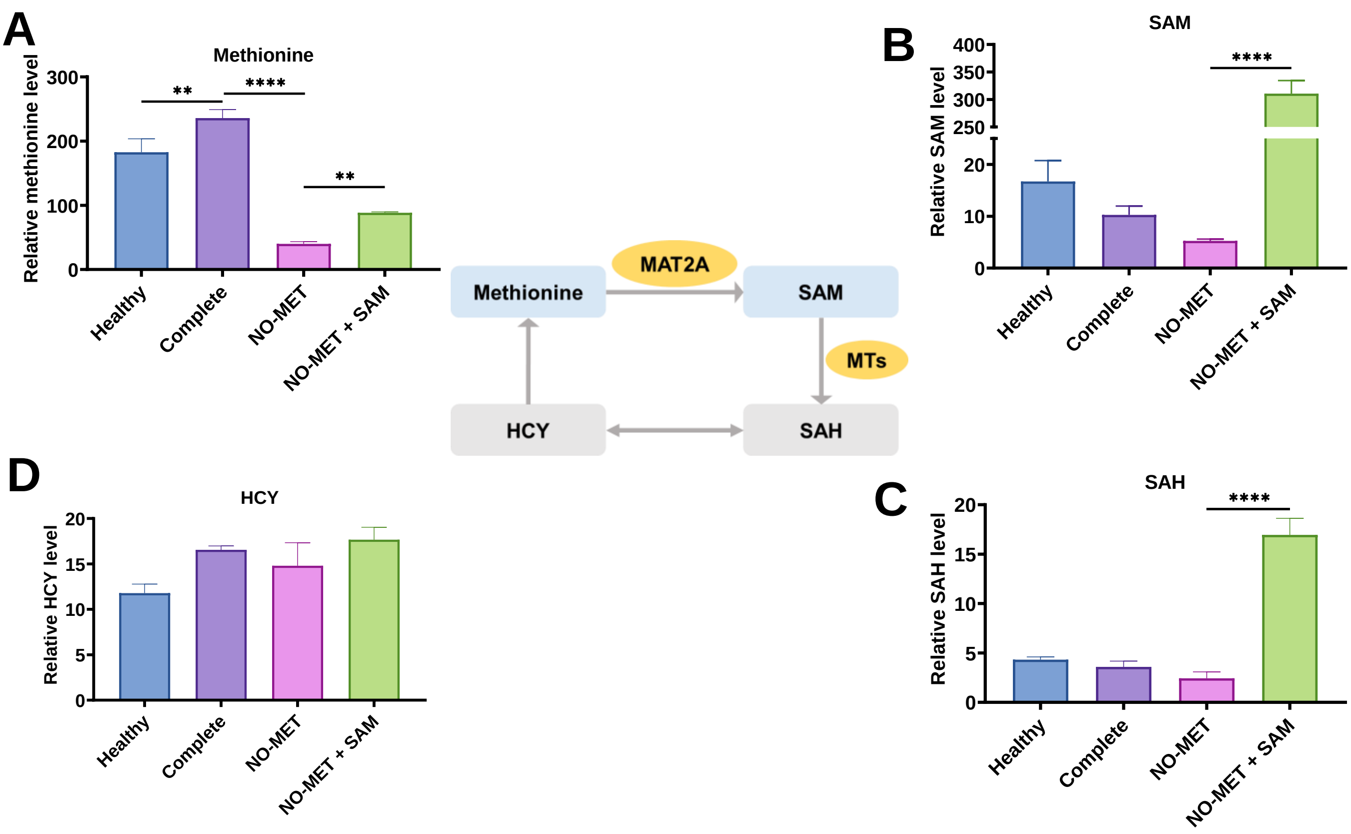

Supplementary Figure 9. Cellular uptake of Methionine and SAM. A-D Healthy PBMCs (Healthy) were treated with complete media. Primary R/R AML blasts were treated with complete media (Complete), methionine-depleted media (NO-MET), or methionine-depleted media supplemented with 500 μM SAM (NO-MET + SAM) for 24h, and the intracellular methionine, SAM, SAH and HCY levels of were measured through mass spectrometry (n=3). $^{ns}p > 0.05$, $^{*}p \leq 0.05$, $^{**}p \leq 0.01$, $^{***}p \leq 0.001$, $^{****}p \leq 0.0001$.

S10

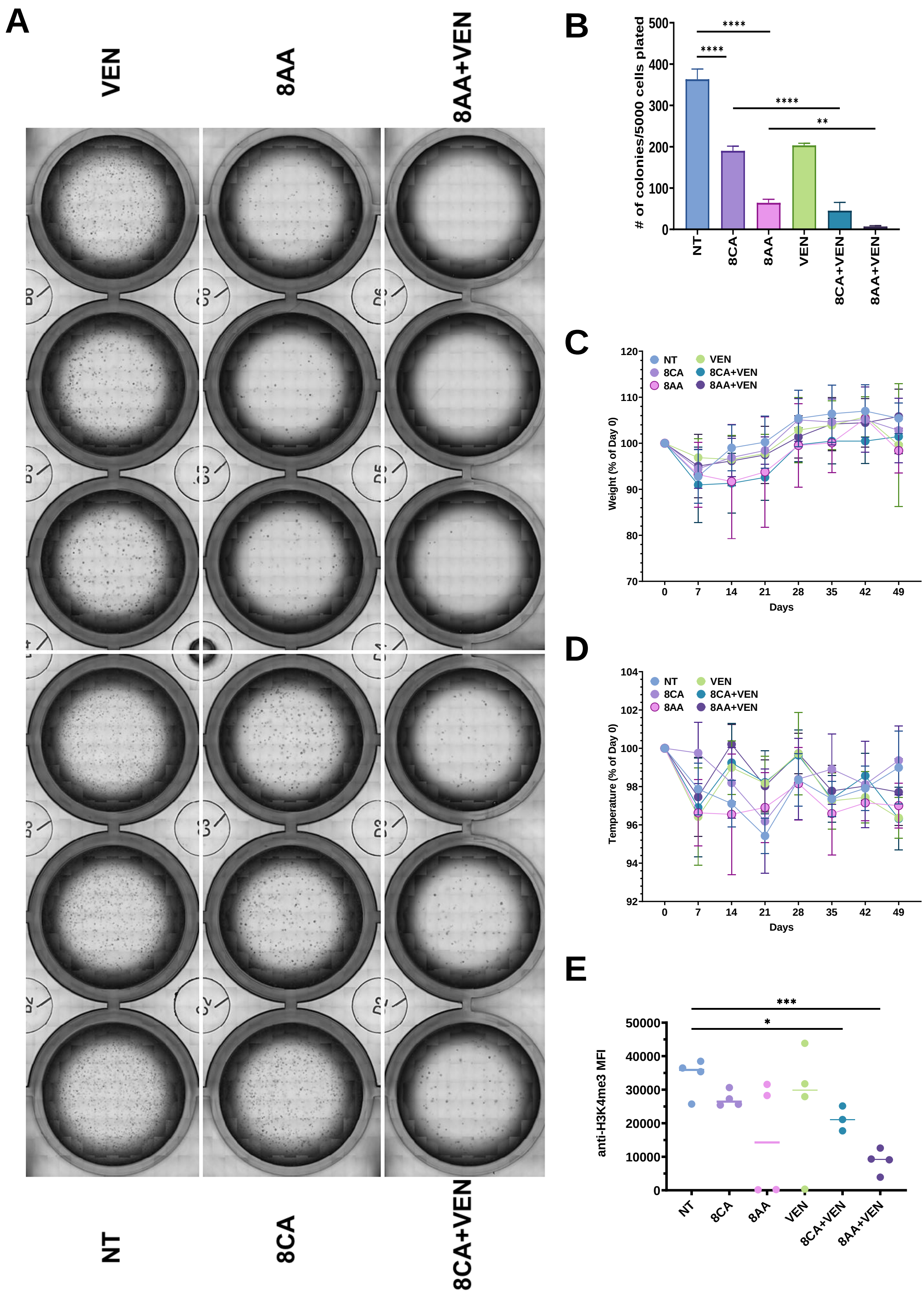

A
VEN
8AA
8AA+VEN
NT
8CA
8CA+VEN
B
# of colonies/5000 cells plated
****
****
****
**
NT
8CA
8AA
VEN
8CA+VEN
8AA+VEN
C
NT
8CA
8AA
VEN
8CA+VEN
8AA+VEN
Weight (% of Day 0)
Days
D
Temperature (% of Day 0)
Days
E
anti-H3K4me3 MFI
***
*

Supplementary Figure 10. 8CA/8AA and VEN synergistically target R/R AML blasts. A-B AML blasts were pretreated with vehicle (NT), 1 μM 8CA/8AA, 0.02 μM VEN or their combinations for 24h, and then cultured in 3D media at the same density to form colonies. The colonies were counted using microscopy and quantified (n=3). **C-D** The body weight and temperature of mice receiving different treatments were monitored. **E** Histone methylations (represented by MFI of anti-H3K4me3) in the mononuclear spleen cells isolated from each group after sacrifice were measured (n≥3). $^{ns}p > 0.05$, $^{*}p \leq 0.05$, $^{**}p \leq 0.01$, $^{***}p \leq 0.001$, $^{****}p \leq 0.0001$.

Supplementary Methods

RNA-Seq

RNA-Seq reads were trimmed to remove sequencing adapters using Trimmomatic[29] and polyA tails using FASTP[30]. The processed reads were mapped back to the human genome (hg19) using STAR software (v. 2.6.0.a)[31]. The HTSeq software (v.0.11.1) was applied to generate the count matrix, with default parameters[32].

Differential expression analysis was conducted by adjusting read counts to normalized expression values using TMM normalization method in R[33].

Pathway analysis was conducted using GSEAP reranked algorithm implemented in GSEA Desktop program in Java, where a ranked list of full genes according to their log2 fold change and p-values was provided[29, 32]. The top 10 downregulated/upregulated pathways were analyzed through jVeen platform[34].

Ribosomal Profiling

We followed the protocol as previously reported[35]. Briefly, the cells were lysed, and centrifuged at 16,000g for 15 min to collect the clear supernatant. The lysate was then added on top of a 5%-to-50% sucrose gradient, which was prepared using a Gradient Maker (BioComp). Subsequently, the samples were centrifuged at 28,000 rpm for 3 h at 4°C using an Optima L-100 XP Ultracentrifuge to separate the components of the lysate based on their density. Then the sample was fractionated into 20 fractions and analyzed using a Gradient Station (BioComp) combined with an ECONO UV monitor (Bio-Rad) and a Gilson FC203B fraction collector (Mandel Scientific). RNA was purified from all fractions obtained from the sucrose gradient separation. Purified RNA samples with sufficient concentration were subjected to RT-qPCR analysis to measure METTL16 expression.

Cell free methylation system

Briefly, recombinant human METTL16 protein (Active Motif), SAM (Sigma-Aldrich), and RNA oligo (5'-UGUUGGCGUAGGCUACAGAGAAGCCUUCA-3', IDT) were incubated with 8CA/8AA or their metabolites 8-amino-ATP or 8-chloro-ATP at 37°C for 3-4 h. Subsequently, the RNA was purified and digested using nuclease P1 (Sigma) followed by dephosphorylation using FastAP Alkaline Phosphatase (Fisher Scientific). The samples were then denatured at 65°C for 10 minutes, and the supernatant was collected by centrifugation and analyzed by triple-quadrupole tandem mass spectrometry.

Isothermal dose-response fingerprint (ITDRF) analysis

ITDRF was performed to investigate the direct binding between 8CA/8AA and METTL16 protein[39]. In detail, AML cell lysis (Input) was incubated with 0 uM, 1 uM, 10 uM and 100 uM of 8CA/8AA for 1 h at room temperature. Then the system was heat shocked at 55°C for 3 min, immediately cooled at room temperature, and centrifuged at 20000g for 20 min at 4°C to collect the supernatant. XT sample buffer (Bio-Rad) were added and then the samples were heated to 100°C for 10 min for further blotting analysis.

Mass spectrometry

For quantitative analysis, calibration curves were prepared by spiking methionine, homocysteine (hCys), SAH and SAM (Sigma, St. Louis, MO) over a concentration range of 0 to 10 µM in water. The calibration curve standards, mouse plasma, and cell pellets were subjected to metabolite extraction using 100 µL of water: 300 µL of solvent mixture (methanol: acetonitrile, 3:1, v/v) spiked with 500 nM d3-methionine, d4-SAH, d3-SAM isotopically labelled internal standards (Toronto Research Chemicals, Toronto, ON). For plasma, 20 to 30 µL were diluted with water to a total volume of 100 µL, whereas cells were directly suspended into 100 µL of water for metabolite extraction. The cell pellets were vortexed for 5 s followed by four 30 sec freeze-thaw cycles for

cell lysis. All the samples were vortexed for 5 s and incubated at -20°C for 10 min for protein precipitation. Samples were then centrifuged at 15,000 rpm for 15 min at 4°C. The supernatants were directly analyzed using multiple reaction monitoring (MRM) on a Waters TQD mass spectrometer using a H-Class UHPLC system (Waters Corporation, Milford, MA) using Acquity UPLC BEH-amide column (1.7 µM, 2.1X100 mm, Waters). The separation was performed using solvent A (95% Acetonitrile with 10 mM ammonium acetate and 0.1% acetic acid) and solvent B (5% acetonitrile with 10 mM ammonium acetate and 0.1% acetic acid) with a gradient decreasing from 99% to 35% A in 4 min, 95% A for 2.5 min followed by 99% A for 2.5 min. The following MRM transitions were surveyed: methionine (*m/z* 150.0 < *m/z* 104.0), hCys (*m/z* 136.0 < *m/z* 90.0), SAH (*m/z* 385.2 < *m/z* 136.4), SAM (*m/z* 399.2 < *m/z* 250.0), d3-methionine (*m/z* 153.0 < *m/z* 107.0), d_4-SAH (*m/z* 389.2 < *m/z* 136.4), d_3-SAM (*m/z* 402.2 < *m/z* 250.0). For hCys, d3-methionine was used as a surrogate internal standard for quantitation. All the analytes exhibited excellent linearity with a coefficient of determination (R^2) ≥ 0.98.

Lentiviral transduction and stable cell line

For lentiviral generation, the envelope plasmid pMD2.G and the packaging plasmid psPAX2 were obtained from Addgene (12259 and 12260, provided by Dr. Didier Trono). MAT2A expressing plasmid pCW57.1-MAT2A was provided by Dr. David Sabatini (Addgene 100521)[33]. 293T producer cells were transfected with plasmids pMD2.G, psPAX2 and pCW57.1-MAT2A by DNA transfection reagent (Lipofectamine LTX; Invitrogen). The supernatant containing lentiviral particles was collected 48 h after transfection. MV4-11 cells were transduced with lentiviral particles and treated with Blasticidin for selection 48h post transduction. After Blasticidin selection for 72h, cells were seeded in 96-well plates at a density of 100 cells or 30 cells/well for single clone selection. Single clones were picked for cell expansion. A stable cell line that constitutively expressed MTA2A (MV4-11-OE) was established, and overexpression of MAT2A was confirmed by Western blot.

mRNA dot blotting

RNA was extracted from cells using RNeasy kit (Qiagen), then enriched for mRNA using Dynabeads Oligo $(dT)_{25}$ (ThermoFisher Scientific) and spotted onto Hybond nylon membranes using dot blot apparatus (Bio-Rad) and crosslinked under UV light.
